# Surveillance and molecular characterization of banana viruses associated with *Musa* germplasm in Malawi

**DOI:** 10.1371/journal.pone.0306671

**Published:** 2026-01-29

**Authors:** Johnny Isaac Gregorio Masangwa, Nuria Fontdevila Pareta, Philemon Moses, Eva Hřibová, Jaroslav Doležel, Isaac Fandika, Sebastien Massart

**Affiliations:** 1 Integrated and Urban Plant Pathology Laboratory, Terra-Gembloux Agro-Bio Tech, University of Liege, Gembloux, Belgium; 2 Department of Agricultural Research Services, Bvumbwe Agricultural Research Station, Thyolo, Malawi; 3 Center for Health, Agriculture and Development Research and Consulting, Blantyre, Malawi; 4 Institute of Experimental Botany, Centre of Plant Structural and Functional Genomics, Olomouc, Czech Republic; 5 Department of Agricultural Research Services, Kasinthula Agricultural Research Station, Chikwawa, Malawi; University of Wyoming, UNITED STATES OF AMERICA

## Abstract

Malawi has diverse local banana germplasms that are preferred by its population. However, the epidemics of banana bunchy top disease (BBTD), caused by the banana bunchy top virus (BBTV) is wiping out the preferred germplasms and limiting their cultivation. A survey was conducted to characterize banana germplasm and evaluate the presence, incidence and prevalence of banana viruses. PCR products from infected germplasm were sequenced and aligned for each detected virus to construct a phylogenetic tree. BBTV, banana mild mosaic virus (BanMMV) and six banana streak virus (BSV) species were detected in Malawi. Malawi’s BBTV isolates belonged to the Pacific Indian Ocean group, and BanMMV isolates clustered to three sub-branches. The six BSV species detected in Malawi belonged to clade 1. Among the genetic groups of *Musa*, the characterized banana germplasms belonged to AA, AAA, AAB, and ABB groups with some germplasms being unique compared to those already genotyped. The ABB group was dominant in Malawi and was significantly more often infected by BSV species (possibly originating from endogenous viral sequences), while BBTV and BanMMV infected the AAA and AAB group more frequently, respectively. The primary source of banana planting materials was banana propagule exchange among relatives which posed a higher risk of spreading virus diseases. The survey underlined the importance of establishing a banana seed industry and implementing policies that promote farmers’ access to virus-tested planting materials, ultimately helping to prevent future virus epidemics.

## Introduction

Banana is an herbaceous plant belonging to the *Musa* genus and the *Musaceae* family [1]. It is ranked as the fourth most important food commodity in the world and represents a staple food and cash crop for millions of people in many developing countries [1,2]. The crop possesses great genetic diversity in terms of its genomic groups based upon the inter- or intra-specific hybridization of *Musa acuminata* and *M. balbisiana* species. The hybridization resulted in the development of AA, AB, AAA, AAB, ABB, AABB, AAAB, or ABBB genotypes [3]. Even though no African country is among the top five world producers of banana, countries such as Uganda, Rwanda and Cameroon have the highest per capita annual consumption, exceeding 200 kg and providing up to 25 percent of the daily calorie intake [4]. Banana is also an important food and cash crop in Malawi. Over 30% of Malawi’s population depends on banana cultivation for their livelihoods. In Malawi, like in many parts of the world, a large proportion of banana fruits are produced in small plots or backyard gardens [5,6]. A study conducted in the early 2000s revealed that in Malawi’s major banana growing districts, 50% of farmers’ income came from banana [7] despite the presence of constraints such as drought, poor soil fertility, poor management, pests, and diseases [8].

Nevertheless, the situation dramatically changed by 2015, as 90% banana plantations in the southern part of Malawi were wiped out by banana bunchy top disease (BBTD) caused by the banana bunchy top virus (BBTV) [9]. BBTV is a member of the genus *Babuvirus* and the family Nanoviridae whose isolates are divided into two major groups: the Pacific–Indian Oceans (PIO) group and South-East Asian (SEA) group. The latter group is confined to the Asian region while the former is widely distributed [10,11]. According to Mikwamba *et al*. [12], the worst economic losses inflicted on many smallholder farmers’ livelihoods due to BBTD were in the Thyolo district in Southern Malawi. Currently, BBTD is also destroying banana plants in Karonga district, which is one of the major banana and plantain growing districts in northern Malawi [13], threatening the survival of banana industry [14]. The Food and Agriculture Organization [15] estimated that BBTV alone accounted for almost 40% of losses in banana production in Malawi and Jekayinoluwa and colleagues [16] indicated that this disease has affected 80% of banana producing areas in Malawi. This banana disease has not only affected farmers but also value chain players such as transporters, traders, agro-processers, researchers, micro-finance institutions and consumers [17]. BBTD forced the country to rely upon imports of over 20,000 tons to meet high demand [Ministry of Agriculture of Malawi – unpublished]. The presence of BBTD also led the country to source over 1,045,250 disease-free tissue culture planting materials from other countries [Ministry of Agriculture – unpublished] at an approximate cost of 2million US dollars.

Surveying and collecting local banana landraces in Malawi are vital for safeguarding and harnessing genetic diversity that may offer unique resistance or tolerance to BBTV or future emerging pests or abiotic stresses. For instance, wild *Musa balbisiana* accessions have demonstrated high levels of resistance and even possible immunity to BBTV, offering valuable genetic resources for both conventional and marker-assisted breeding programs [18]. By characterizing, conserving and safeguarding this in-situ diversity, researchers can identify candidate resistance traits and integrate them into breeding pipelines. This will ultimately facilitate enabling the development of locally adapted, disease-resilient planting materials that safeguard farmers’ livelihoods and contribute to the long-term sustainability of banana sector.

Beyond BBTV, several other viruses affect banana and other vegetatively propagated crops [18]. More than 20 virus species are reported worldwide [1]. Banana streak viruses (BSVs), belonging to the genus *Badnavirus* (family Caulimoviridae), cause chlorosis, necrotic streaks, stunting, fruit distortion, smaller bunches and sometimes plant death [19–21]. Currently, nine BSV species are currently recognized by ICTV, including BSOLV, BSMYV, BSIMV, BSGFV, BSUAV, BSUIV, BSUMV, BSVNV, and BSULV, with others tentatively classified [22]. Functional viral genomes of some species (BSGFV, BSIMV, BSOLV) are integrated into the *Musa* B genome [23,24]. Yield losses range from 6–15% depending on cultivar, virus strain, and environment [25,26], though symptoms may sometimes disappear after two years, as shown in Guadeloupe [26]. Banana mild mosaic virus (BanMMV), a ssRNA virus of the genus *Banmivirus* (Betaflexiviridae), is mainly spread via infected planting material and lacks a known natural vector [27,28]. Though not considered a major threat [20], BanMMV can exacerbate cucumber mosaic virus (CMV) symptoms in co-infections [29]. CMV, with a broad host range, is endemic in most banana-growing regions [30] and causes chlorosis, mosaic, heart rot, stunting, and yield decline [30,31]. Banana bract mosaic virus (BBrMV) induces spindle-shaped streaks and mosaic patterns on bracts and leaves [32–34], and is reported in Asia and the Americas [35,36]. Banana viruses spread mainly via vegetative propagation [20,25,37] and by insect vectors: aphids for BBTV [38] and BBrMV [39], and mealybugs such as *Saccharicoccus sacchari*, *Planococcus citri*, *Paracoccus burnerae* and *Dysmicoccus brevipes* for BSVs [40,41].

Beyond BBTV, other viruses can cause problems in banana and other vegetatively propagated crops [19]. So far, only BBTV has been officially reported in Malawi. No study has been done to investigate other banana viruses in the country, their hotspot areas, and prevalence among Malawi’s banana cultivars. Lack of knowledge of other banana viruses may lead to a rapid distribution of infected banana planting materials which could result in an outbreak of other viral diseases. With this in mind, the study was executed to evaluate the presence and incidence of banana viruses in *Musa* crops through a survey and targeted molecular detection of the viruses at country-wide level. Additionally, plot maps of all areas endemic to viruses were created. The relationship between genotypes, source and age of banana mats (the banana plants that arise from a single underground rhizome) and virus infections were investigated. The surveyed area was divided into four regions, referred to as clusters in this study.

## Materials and methods

### Field survey

**Fig 1:**
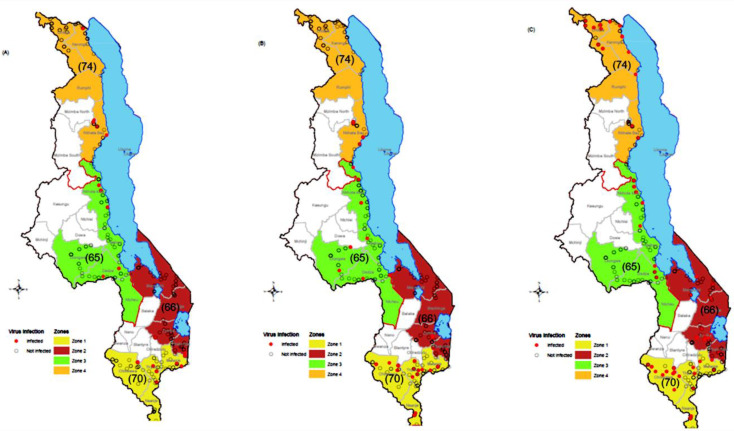
Maps of banana virus (BBTV, BSVs, BanMMV) test results – created by Masangwa et al. **(A–C)** Mapping of the 180 sampled farms throughout Malawi. Each sampling location corresponds to a circle; one or several samples were sampled in each location, resulting in 275 samples for virus testing). The number of samples per zone is indicated between brackets for each zone. A: location of samples tested positive for BBTV (red circle). B: location of samples tested positive for BSVs (red circle). C: location of samples tested positive for BanMMV (red circle).

A survey was conducted in August 2020 across Malawi in four banana cultivation zones (Fig 1A–1C). Mapping of the 180 sampled farms throughout Malawi. Each sampling location corresponds to a circle; one or several samples were sampled in each location, resulting in 275 samples for virus testing. The number of samples per zone is indicated between brackets for each zone. A: location of samples tested positive for BBTV (red circle). B: location of samples tested positive for BSVs (red circle). C: location of samples tested positive for BanMMV (red circle). Zone 1 corresponded to the Southern region districts of Chikwawa, Mulanje, Nsanje and Thyolo; Zone 2 included the Eastern region districts of Phalombe, Machinga, Mangochi and Zomba; Zone 3 was the Centre region with Dedza, Lilongwe, Salima and Nkhotakota districts; and Zone 4 was the North region of Chitipa, Karonga, Rumphi and Nkhata-bay districts. The listed sites laid within latitude −9.597858 S to −17.102709 S and longitude 33.219222 E to 35.814582 E. The areas’ altitude ranged from 48 to 1,665 meters above sea level (masl).

Fourty-five farms, with a minimum distance of 5 km between each other, were visited per cultivation zone (180 farms for four zones). On large fields with a single cultivar, the W sampling pattern was done to sample 5–10 individual mats and to generate a composite sample for this cultivar in this farm. Sometimes, only one plant of a particular variety was present, and it was sampled. For each individual mat, leaf samples from the 3rd uppermost leaf from the apex were collected and dried. If viral symptoms (mosaic or streaks) were observed, the plant was sampled and analyzed independently. Leaf samples with fungal necrotic symptoms were not collected. Collected leaves were dried over silica gel [42].

### Germplasm collection

The task of germplasm collection was conducted simultaneously with leaf sampling during the survey. The banana germplasm collection form and a minimum list of morphological descriptors, adapted from the descriptors for banana (*Musa* spp.) [43], was utilized during the germplasm collection process. Key data collected included the plant family name, species, local name, the meaning of the name, and the language or ethnic group conserving (cultivating) the species. Two suckers of each variety, considered as unique compared to the already collected varieties, were collected with the oral consent of the field owner.

### Targeted virus detection

Fourty milligrams of dried leaf samples were ground in 4 ml of CEB extraction buffer [44] in the Agdia extraction bags (Agdia-EMEA, Soisy-sur-Seine, France) using a tissue homogenizer (Agdia, Elkahart, USA). Leaf samples from reference banana plants known to be infected with various viruses and maintained at GxABT were used as positive controls, while PCR-grade water served as the negative control. Depending on the species of the virus, immuno-capture PCR or immune-capture RT-PCR was done on the collected leaves. Tubes were coated with antibodies as described by De Clerck *et al.* [33], then proceeded by adding 25 µl of plant extracts. The standardized protocols of the *Musa* Germplasm Health Unit (from the University of Liège) described by De Clerck et al. [33] were applied for the detection of viral species infecting banana. Complementary DNAs were prepared for RNA viruses. For DNA viruses (e.g., BSV) DNase I (Invitrogen, stock 277 U/μl) treatment was carried out after immunocapture to eliminate residual DNA. Virus specific PCRs were prepared following the protocol (S1 Table) and amplicons were visualized on TAE buffer (1X) on a 1% agarose gel stained with Gel red® (Biotium).

### Genotyping of Malawi’s banana cultivars

Local *Musa* landraces were collected from across Malawi for molecular characterization. Genomic DNAs of banana germplasms were isolated from the cigar leaf and subjected to microsatellite (SSR) genotyping following the protocol of Christelová *et al*. [45]. The SSR data obtained from Malawi’s germplasms were analyzed together with the *Musa* core set collection [46] using DARwin software [47] and the cladogram was depicted with FigTree v1.4.4 (http://tree.bio.ed.ac.uk/software/figtree/). Ploidy level of local landrace was determined using flow cytometry (FCM) according to Doležel *et al*. [48] and Christelová *et al.* [45].

### Map plotting and data analysis

Data collected during field-survey and from the virus indexing were combined with the administrative boundaries obtained from the local online geodatabase MASDAP (Malawi Spatial Data Platform; http://www.masdap.mw/catalogue/#/?f=dataset), then plotted using ArcMap in ArcGIS 10.5 software [49]. All collected data were subjected to the descriptive analysis of all variables in SPSS v.25 to generate frequency tables showing the prevalence of variables within zones. Normality test of variables was done using the Kolmogorov-Smirnov and Shapiro-Wilk tests (P > 0.05) in the Statistical Package for the Social Sciences (IBM SPSS Statistics, v.28.X) software program (S2A–S2D Table). Normally distributed data were analyzed using the Pearson Chi-square test to investigate the relationship between cultivation zones and factors such as sources of banana mats, banana genotypes, age of mats, and cultivation systems. The strength of associations between variables was determined using the Phi coefficient, as described by Akoglu [49].

The viral prevalence data were not normally distributed and were first screened to remove categories with fewer than five samples. A total of 275 individual samples were collected, all of which were used in the zone and cultivation system analysis. For banana mat age, 270 samples were analyzed, while 254 samples were used for source-of-plant analysis and 256 samples for genotype analysis. Before further analysis, data were subjected to Snedecor and Cochran’s rule (np > 15 and nq > 15) to confirm their approximate binomial distribution (np) and their variance (npq), where n represents the number of samples, p is the probability of infection, and q = (1-p) is the probability of non-infection (S3A–S3E Table). All data that met Snedecor and Cochran’s criteria were analyzed using the non-parametric exact binomial test in R (https://www.r-project.org/, version 4.4.1) within RStudio (https://posit.co/download/rstudio-desktop/, version 2024-06-14). The test was used to examine the effects of sources of mats, banana genotypes, mat age, cultivation systems, and zones on the incidence of detected viruses (1 = present, 0 = absent). Each variable was analyzed separately for each virus. The significance levels between categories of each variable were then assessed using pairwise comparisons with the emmeans package [50].

The Odds Ratios (ORs) were then calculated to measure the association that quantifies how the odds of an outcome (like a disease) change with exposure to a particular factor. The formula that was used was:


$$\text{OR}=\;\frac{\text{Odds}\;\text{of}\;\text{outcome}\;\text{in}\;\text{exposed}\;\text{group}}{\text{Odds}\;\text{of}\;\text{outcome}\;\text{in}\;\text{non}-\text{exposed}\;\text{group}}$$

Confidence Intervals (CIs) which are ranges of values within which the true value of a parameter (like OR) are likely to fall, with a given level of confidence (usually 95%) were computed using the formula:


$$\text{CI}=\;e^{x[In(OR)\pm zxSE\left(In(OR)\right)]}$$

where: e = exponential function (base of natural logarithm); In(OR) = natural logarithm of the odds ratio; Z = z-score corresponding to the desired confidence level of 1.96 for 95% confidence; SE(In(OR)) = standard error of the logarithm of the ratio [53].

### Sequencing and phylogenic comparison of banana viruses present in Malawi

PCR products from samples tested positive for a virus were purified using Qiaquick purification kit (Qiagen) according to the manufacturer’s instructions. The purified PCR products were then sequenced at Macrogen Europe using the specific primers (S1 Table). The sequences used for the phylogenic comparison were the partial DNA-R gene for BBTV, Coat Protein (CP) for BanMMV and ribonuclease H (RNAse H) for BSV from our study; and sequences that were downloaded from NCBI’s Nucleotide repository (S4 Table). The BBTV South Pacific group and the Asian group of isolates were used as references. BSV sequences from three BSV Clades as published by Chabannes *et al.* [24] were used as references for BSV.

Different bootstrap maximum likelihood phylogenic trees, replicated 1000 times, were constructed using MEGA X software (https://www.megasoftware.net) to determine the homology and evolutionary relationships of the viruses in each group (BanMMV, BBTV, and BSV). Using the p-distance method the distances of the isolates were analyzed to understand their evolutionary history.

## Results

### Collection and molecular characterization of banana cultivars

During the survey, 275 leaf samples were collected from 17 districts of Malawi corresponding to four main banana cultivation zones with the following proportions: 25% from Zone 1 (n = 70); 24% from Zone 2 (n = 66); 24% from Zone 3 (n = 65) and 27% from Zone 4 (n = 74). There were variabilities in terms of the numbers of individual samples per zone due to variation in terms of number of cultivars sampled per farm. The study sampled all cultivars that were found at each farm. The collected samples corresponded to 38 banana cultivars (Fig 1A–1C and S5 Table).

Cliquez ou appuyez ici pour entrer du texte.The ABB genotype was the most sampled (58%; n = 159/275) of all the collected leaf samples, whatever the zone (from 46% to 74%), followed by AAA at 25% (n = 68/275) (S1 Fig and S6 Table) [45,46]. Cliquez ou appuyez ici pour entrer du texte.

**Fig 2:**
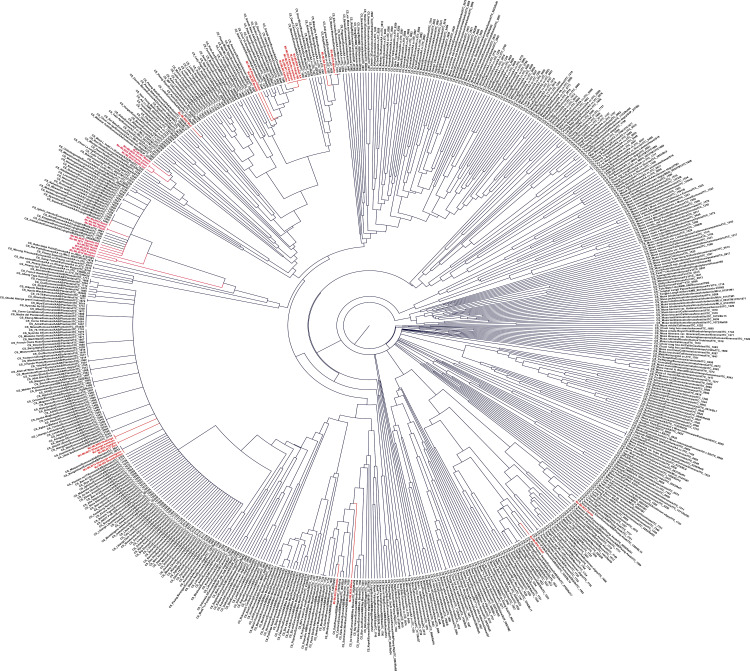
Cladogram of Malawian and already genotypes characterized *Musa* germplasm in the ITC Bioversity Genebank. Cladogram of Musa germplasm based on SSR profiling [46]. It includes the 33 Malawi germplasms genotyped (in red) and the core collection (591 germplasms) of the International Transit Center Gene bank (Alliance Bioversity International-CIAT; in black).

Banana core subset of genotypes of the international germplasm collection from Bioversity International contained 591 genotyped germplasms (in black), representing individual species/subspecies and subgroups of *Musa*, enabling a detailed genetic characterization of unknown banana genotypes [46,51,52]. The molecular characterization by SSR typing of 33 genotypes from Malawi (in red) revealed that Malawi’s banana landrace germplasms belonged to 4 genomic groups AA, AAA, AAB and ABB 4 (Fig 2 and S6 Table). Fig 2. Cladogram of Malawian and already genotypes characterized *Musa* germplasm in the ITC Bioversity Genebank Cladogram of Musa germplasm based on SSR profiling [46]. It includes the 33 Malawi germplasms genotyped (in red) and the core collection (591 germplasms) of the International Transit Center Gene bank (Alliance Bioversity International-CIAT; in black). The SSR typing revealed that twenty-one (in red) Malawi *Musa* germplasm belonged to AAA group, seven (in red) belonged to Plantain and Silk groups (AAB genome). Three Malawi germplasms represented Monthan and Pisang Awak banana groups with the ABB genome. Two Malawi germplasms (Mthwika and Suweshi) were diploid AA Mchare bananas, one of the progenitors of triploid Cavendish and Gros Michel edible banana clones. Compared to the existing diversity (591 germplasm – in black), four Malawian germplasms (in red) were unique: Mabere (ABB) was distant from other ABB germplasms while Munowa; Kapeni and Ndifu germplasms were also distant from other AAA genotypes [46].

### Survey summary: Source of plant, age and cultivation system

The source of banana planting materials was investigated to understand Malawi’s banana seed system. This study identified six sources of banana mats: exchange among relatives, floods (suckers found in fields after flooding), government, Church/non-governmental organizations (NGO), own-field (Seed serve) and purchase (S5 Table). Prevalence of source of mat was evaluated and exchange of propagules among relatives was by far the most popular (70%, n = 191/275) source of mat, while NGO/Church was the lowest (0.4%, n = 1/275). Surprisingly, the results also unveiled floods (6%; n = 7/275) as a source of mat.

The analysis of the age of the mat revealed that 47% (n = 130/275) of the total banana mats were over 6 years old, followed by 39% of mats of 1–3 years old (n = 107/275) (S7 Table).

The type of banana cultivation systems corresponded to monocropping (39% - n = 107) or mixed cropping (61% - n = 168) (S8 Table).

### Exploring inter-variable dynamics

#### Associations between banana cultivation zones and other variables.

The banana cultivation zone was very highly significantly related to the banana cropping systems (χ² = 20.942, df = 3, P ≤ 0.001, φ = 0.276). Banana cultivation Zone 3 had the highest number of mats under mono-cropping (57% - n = 37) (S8 Table). The other zones recorded high numbers of banana mats under mixed cropping (from 51% to 74%).

There was a highly significant relationship between banana cultivation zones and ages of banana mats (χ² = 21.286, df = 3, P ≤ 0.002, φ = 0.278). For example, mats of 1–3 years old recorded 57% (n = 40/70) of the total mats within Zone 1 (S7 Table) while in other zones, mats of over 6 years old were the most prevalent: 45% (30), 49% (32) and 54% (40) in Zones 2, 3 and 4, respectively.

Genotype was another parameter that presented a significant relationship (χ² = 14.363, df = 6, P ≤ 0.026, φ = 0.237) with cultivation Zone (S9 Table). The AAA genotype was more prevalent in Zone 1 (36% n = 24/66). The AB genotype was only present in Zone 4 (7% - n = 74) (S10 Table). The results showed no significant relationship (χ² = 23.250; df = 15; P = 0.079; φ = 0.293) between banana cultivation zones and source of mat (S11 Table).

#### Relationships between other variables.

A very highly significant relationship (χ² = 24.34; df = 4; P ≤ 0.001; φ = 0.299) was observed between banana mat age and the source of banana mat (S12 Table). Sharing contributed over half (59/95) of the total mats of 1–3 yrs old and 83% (103/124) of banana mats of over 6 yrs old. Within purchase as a source, it was observed that 74% (23/31) of banana mats were between 1–3 yrs old compared to 23% (n = 7/31) that were over 6 yrs. Another significant relationship was observed between banana cropping systems and banana genotypes (χ² = 7.19; df = 2; P = 0.027; φ = 0.168) (S13 Table). Monocropping recorded the highest prevalence (73%) of ABB genotype compared to 57% registered by the mixed cropping. The opposite was observed for the AAA genotype, where mixed cropping had the highest (30%) prevalence than 18% in mono cropping.

There was no significant relationship (S14–S17 Tables) between banana cropping system and age of mats (χ² = 5.648; df = 2; P = 0.059; φ = 0.143); between cropping system and source of banana mats (χ² = 3.729; df = 4; P = 0.444; φ = 0.121), between banana genotypes and age of mats (χ² = 3.729; df = 4; P = 0.444; Phi = 0.121 and source of mat (χ² = 5.521; df = 4; P = 0.238; φ = 0.152).

#### Virus detection.

The virus testing showed that 41% (n = 113/275) of the plants tested positive to at least one banana virus. BanMMV, BBTV and BSVs were detected in all the four zones (Fig 1A–1C) with a prevalence of 12% (n = 32), 8% (n = 23), and 23% (n = 64) respectively. No sample tested positive to CMV or BBrMV. The prevalence of each BSV species was 8% for BSOLV and BSIMV, 5.5% for BSMyV, 5% for BSGFV, 2% for BSCaV and 0.4% for BSLacV (S18 Table).

Among the positive samples, 24 (9% - from zones 1, 3 and 4) tested positive to multiple virus infections (S2 Fig and S19 Table). Quadruple infection was detected on 2 ABB samples from Karonga district (Zone 4). BBTV and three species of BSVs (BSMYV, BSGFV and BSOLV) were detected in Zanzibar cultivar and quadruple infections of BSV (BSCAV; BSGFV; BSIMV and BSMYV) was detected in Zanda cultivar. ABB genotype from five sites (two in Zone 1 and three in Zone 3) tested positive to BanMMV, BBTV and one BSV species (BSIMV, BSOLV, BSGFV or BSMYV).

#### Geographical spread of banana viruses in Malawi.

BanMMV, BBTV and at least one species of BSV were detected in the districts of Chikwawa, Mulanje, Thyolo (Zone 1); Phalombe, Thyolo, Zomba (Zone 2); Dedza, Nkhotakota (Zone 3) and Nkhatabay (Fig 1A–1C). BanMMV was the only virus detected in Nsanje, Lilongwe and Salima while BSV was the only virus detected in Chitipa, Machinga and Mangochi. BBTV was detected in all zones although it was not detected in all districts within zones. The districts with BBTV presence were Chikwawa, Dedza, Karonga, Mulanje, Nkhotakota, Nkhatabay, Phalombe, Thyolo and Zomba (Fig 1A). BBTV was detected for the first time in the northern part of Karonga district near Tanzania and Phalombe.

### Factors influencing virus prevalence

#### Effect of Zones on the prevalence of banana viruses.

The zone of cultivation had a significant effect on the prevalence of BanMMV (P = 0.003, df = 3; Table 1). Twenty-nine percent (n = 20/70) of banana samples from Zone 1 tested positive for BanMMV, whereas prevalence was only 6% (n = 4/66) and 9% (n = 7/74) in Zones 2 and 4, respectively (Table 1). No significant association (P > 0.05) was found between cultivation zone and the prevalence of BBTV or BSV, except for Zone 3 in the case of BSV (P = 0.041).

**Table 1:** Effects of banana cultivation zones on the prevalence of three viruses: BanMMV, BBTV and BSV (positive to at least one BSV species).

Zone	Number of mats	Virus	% prevalence (n)	Odds	95% CI	P value
Zone 1	70	BBTV	14% (10)	Reference		
		BanMMV	29% (20)	Reference		
		BSV	29% (20)	Reference		
Zone 2	66	BBTV	8% (5)	0.49	0.18 - 1.35	0.219
		BanMMV	6% (4)	0.16	0.05–0.50	0.002**
		BSV	21% (14)	0.61	0.28–1.36	0.230
Zone 3	65	BBTV	9% (6)	0.61	0.25 - 1.47	0.367
		BanMMV	15% (10)	0.45	0.20–1.06	0.069
		BSV	14% (9)	0.40	0.17 - 0.96	0.041*
Zone 4	74	BBTV	11% (8)	0.73	0.31 - 1.70	0.530
		BanMMV	9% (7)	0.26	0.10–0.67	0.005**
		BSV	30% (22)	1.06	0.53 - 2.13	0.8785

Columns represent zone, number of mat, virus, prevalence (number), P = P value, 95% CI = 95% confidence interval. *: significantly different (P < 0.05); ** very significantly different (P < 0.01); ***very highly significantly different (P < 0.001).

#### Impact of banana genotype on virus prevalence.

Group AA was not included in the analysis because the small number of samples would have limited the statistical power of the test. Binomial test results indicated that genotype had a highly significant effect on BBTV (P < 0.001) and BSV (P = 0.003) prevalences (Table 2). The AAA genotype showed a significantly higher BBTV prevalence (26%) compared to ABB (5%, P < 0.001) and AAB genotypes (7%, P ≤ 0.052) (S2 Table). In contrast, AAA genotypes exhibited a significantly lower BSV prevalence (11%, n = 7/65) than ABB (28%, P = 0.0004) and AAB genotypes (28%, P ≤ 0.038). No significant effect of genotype was observed on BanMMV prevalence (P = 0.621).

**Table 2:** Effects of banana genotypes on the prevalence of three viruses: BanMMV, BBTV and BSV.

Genotype	Number of mats	Virus	% Prevalence (n)	Odds	95% CI	P
AAA	65	BBTV	26% (17)	Reference		
		BanMMV	14% (9)	Reference		
		BSV	11% (7)	Reference		
AAB	28	BBTV	7% (2)	0.22	0.05 - 1.01	0.052
		BanMMV	18% (5)	1.35	0.41 - 4.46	0.621
		BSV	28% (8)	3.31	1.06. - 10.37	0.038*
ABB	163	BBTV	5% (8)	0.15	0.06 - 0.39	0.000***
		BanMMV	16% (26)	1.18	0.51 - 2.73	0.691
		BSV	28% (45)	3.16	1.31 - 7.61	0.008**

Columns represent genotype, number of mat, virus, prevalence (number), P = P value, 95% CI = 95% confidence interval. *: significantly different (P<0.05); ** very significantly different (P<0.01); ***very highly significantly different (P<0.001).

The impact of genotype on virus prevalence was evaluated within each banana cultivation zone. Differences in BSV prevalence between genotypes were observed only in Zones 1 and 4. In these zones, genotype ABB showed the highest proportion of BSV-positive samples, with 47% (n = 18/38) in Zone 1 and 41% (n = 13/32) in Zone 4, compared to other genotypes (S10 Table). BBTV prevalence between genotype were observed in Zones 1, 2 and 4 in which genotype AAA had highest proportions 23% (n = 6/27), 36% (n = 5/14) and 35% (n = 6/17) respectively, compared to genotype ABB.

#### Effect of source of mat on virus prevalence.

Due to low statistical power, categories such as floods, government, and NGO/Church were excluded from the dataset prior to analysis. Results showed that source of mat had a significant effect on BBTV prevalence. Mats from own-fields had the lowest prevalence (3%) compared to from other sources (OR = 6.46, 95% CI = 0.04–2.69, P < 0.001). Source of mat had no significant effect on BanMMV (P = 0.066; OR = 2.39, 95% CI: 0.95–6.02) or BSV (P = 0.160; OR = 0.44, 95% CI: 0.14–1.36) (Table 3). Exchange of propagules showed the highest prevalence for the three detected viruses: BBTV (16%, n = 19/117), BanMMV (22%, n = 26/117), and BSV (41%, n = 48/117), compared with other sources where maximum prevalence reached only 3.4%, 11.1%, and 5.1%, respectively (Table 3).

**Table 3:** Effects of sources of banana mats on the prevalence of three viruses: BanMMV, BBTV and BSV.

Source	Number of mats	Virus name	% prevalence (n)	Odds	95% CI	P
Sharing	193	BBTV	10% (19)	Reference		
		BanMMV	14% (26)	Reference		
		BSV	25% (48)	Reference		
Own-field	29	BBTV	3% (1)	6.46	0.04–2.69	0.000***
		BanMMV	27% (8)	2.39	0.95 - 6.02	0.066
		BSV	21% (6)	0.78	0.30 - 2.02	0.61
Purchase	31	BBTV	13% (4)	1.36	0.42–4.12	0.535
		BanMMV	10% (3)	0.72	0.20 - 2.51	0.60
		BSV	13% (4)	0.44	0.14 - 1.36	0.16

Columns represent source, number of mat, virus, prevalence (number), P = P value, 95% CI = 95% confidence interval. *: significantly different (P<0.05); ** very significantly different (P<0.01); ***very highly significantly different (P<0.001).

#### Ages of banana mat and crop production systems have no impact on virus prevalence.

Age of mat did not impact the BBTV (P = 0.899, OR = 1.49, 95% CI: 0.40–5.59); BanMMV (P = 0.385, OR = 0.74, 95% CI: 0.10–1.32); and BSV (P = 1.000, OR = 1.77, 95% CI: 0.48–6.54) prevalences (S20 Table).

Cultivation systems had no effect on the prevalence of BBTV (P = 0.382, OR =,1.69 95% CI: 0.72–3.99), BanMMV (P = 0.498, OR = 0.79, 95% CI: 0.40–1.6) and BSV (P = 0.625, OR= 0.69, 95% CI: 0.30–1.58) in Malawi (S21 Table).

#### Diversity of banana viruses in Malawi.

**Fig 3:**
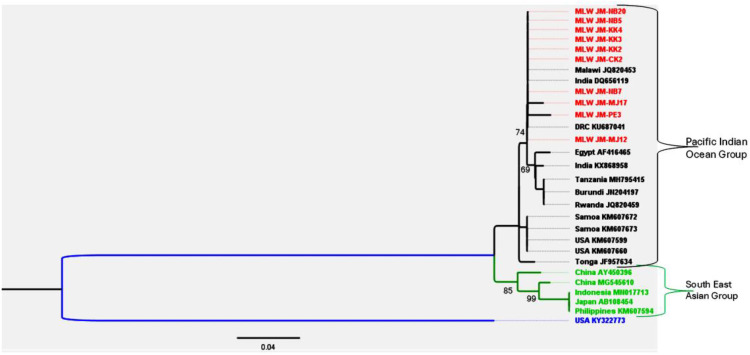
The BBTV partial DNA-R sequences phylogenetic tree. This BBTV phylogenetic tree constructed using Maximum Likelihood method and General Time Reversible model in MEGA from partial DNA-R sequences from this study (in red) and reference sequences from nt database (Genbank – NCBI, in black for Pacific Indian group, in blue for Hawaii and in green for South-East Asian group).

PCR amplicons generated for BBTV (n = 13), BanMMV (n = 16), and BSV (n = 13) were sequenced and deposited in GenBank (S22 Table). The genetic diversity of these viruses was then assessed at a country-wide scale. Fig 3. Maximum-likelihood phylogenetic tree of BBTV isolates based on partial DNA-R sequences. The tree was constructed using the Maximum Likelihood method with the General Time Reversible model in MEGA. Sequences from this study are shown in red, while reference sequences retrieved from the NCBI GenBank database are shown in black (Pacific Indian Ocean group), blue (Hawaii group), and green (South-East Asian group). Phylogenetic analysis of BBTV (Fig 3) showed that all isolates from this study clustered within the Pacific Indian Ocean (PIO) group and were 100% identical at the nucleotide level to a Malawi isolate (JQ820453) obtained in 2012. The isolates also shared 99.1% nucleotide identity with additional isolates from Malawi (ON934241), Tonga (JF957634), Tanzania (MH795415), and Rwanda (JQ820459).

The BSV phylogenetic tree indicated that all isolates from this study clustered within Clade 1 (S3 Fig). BSV partial RNase H Gene Maximum Likelihood phylogenetic tree. BSV Maximum Likelihood method BSV phylogenetic tree constructed using Maximum Likelihood method and Jukes-Cantor model in MEGA from partial RNase H Gene sequences from this study (in red) and reference sequences from nt database (Genbank – NCBI) in black for Clade 1; blue for Clade 2 and green for Clade 3. The detection was validated by sequence analysis for BSIMV, BSMyV, BSOLV and BSGFV but not for BSV-Lac and BSCAV (due to the lack of appropriate biological material). Isolates JM-CK5, JM-CK6, JM-CK7, JM-CP11a, JM-CP11b, and JM-KA2a shared 99% nucleotide identity with the Kenyan BSIMV isolate NC_015507 and 100% identity with the Clade 1 BSIMV isolate AJ002234. Isolates JM-CP12 and JM-KA2b showed 89.9% and 98.5% nucleotide identity, respectively, with BSGFV isolate AY493509. Two isolates, JM-DZ4 and JM-DZ8, corresponded to BSMYV and shared 92.3% identity with isolate AY805074. Finally, isolate JM-CK8 shared 90.7% nucleotide identity with two Cuban BSOLV isolates (FJ527427 and AJ002234).

For BanMMV, four clusters of isolates were identified (S4 Fig**).** BanMMV partial Coat proten maximum likelihood phylogenetic tree. BanMMV maximum Likelihood method phylogenetic tree constructed using Jukes-Cantor model in MEGA from partial Coat protein (CP) from this study (in red) and reference sequences from nt database (Genbank – NCBI) in black. oat protein maximum likelihood phylogenetic tree. Isolates JM-DZ18, JM-KK7, JM-NB25, JM-NE6, and JM-KK10, originating from four different cultivation zones, were 100% identical at the nucleotide level and showed 98.8% identity with the Australian isolate NC_002729. Similarly, isolates JM-CK5, JM-LL10, JM-LL13, JM-MJ11, and JM-TO18, collected from two zones, were 100% identical to each other and to isolate NC_002729. Isolate JM-MJ21 formed a distinct cluster, sharing 92.6% nucleotide identity with the Malaysian isolate FJ179164. Pairwise analysis further revealed that isolates JM-PE13 and JM-NB9 shared 91.4% and 93.8% nucleotide identity, respectively, with Papua New Guinea isolate MT872725.

## Discussion and conclusion

Plant disease surveillance is essential to safeguard food security, as epidemics can severely impact both agricultural productivity and human well-being [53,54]. This study mapped the prevalence and diversity of banana viruses across landraces in Malawi, providing critical insights into disease hotspots, epidemiological drivers, and the role of germplasm diversity. By combining virus detection with farmer interviews and genotype characterization, the results offered a comprehensive picture of both biological and social factors influencing virus spread.

Overall, 41% of banana plants were infected by at least one virus, with BanMMV (12%), BBTV (10%), and six BSV species (23%) detected. The most common BSV species were BSOLV and BSIMV (8% each), while CMV and BBrMV were not detected, suggesting minimal or absent prevalence. Compared with earlier reports of BBTV prevalence in Malawi (46% in 2009), the lower prevalence observed here (10%) may reflect regional variation or the effect of eradication campaigns. However, detection of BBTV in new areas such as Phalombe highlights its ongoing spread into previously unaffected zones. Importantly, co-infections at field level (9% of samples) were detected. Mixed infections can aggravate disease symptoms and complicate management strategies [55–58]. It should be noted, however, that some samples were composite field samples, and verifying co-infection at the individual-plant level will be necessary to fully interpret these patterns.

Spatial analyses revealed that BanMMV prevalence was higher in Zone 1, likely due to frequent exchange of asymptomatic planting materials to rebuild the banana industry. By contrast, BBTV and BSV prevalence showed no strong association with zones, reflecting their widespread dissemination through informal propagule exchange and, potentially, limited efficacy of removal of infected plants [12,13]. Genotype-specific effects were evident: AAA genotypes were more susceptible to BBTV, while B-genome–containing genotypes (AAB, ABB, AB) exhibited higher BSV prevalence (26–33% versus 7% in AAA), consistent with the activation of endogenous BSV sequences integrated into the Musa B genome [22,27,33,59–61] The asymptomatic infection of ABB cultivars such as Zanda and Kholobowa with BBTV raises concerns, as these could act as hidden inoculum sources that undermine eradication programs.

Phylogenetic analyses supported these epidemiological findings: BBTV isolates clustered in a single clade with the Pacific–Indian Ocean group, BanMMV isolates grouped with Australian, Malaysian, and Papua New Guinean isolates, and BSV isolates clustered with Kenyan strains. The diversity of Musa germplasm in Malawi, four genome groups (AA, AAA, AAB, ABB), also shaped virus dynamics. The dominance of ABB cultivars, likely due to their relative tolerance to BBTD compared with susceptible AAA varieties, reflects farmer preferences shaped by past epidemics. However, farmer-driven naming practices and widespread informal seed exchange might perpetuate virus spread and complicate germplasm management.

From a management perspective, this study underscores the urgent need to strengthen surveillance and formalize seed systems in Malawi. Informal propagule exchange among relatives remains the primary source of planting material, but it can also be the main driver of virus dissemination. Asymptomatic but infected germplasm must be identified through indexing to prevent hidden sources of inoculum from sustaining epidemics. Policies ensuring that only certified, virus-tested planting materials are distributed, coupled with registration of seed producers and routine inspection by plant health authorities, are essential.

Finally, while CMV and BBrMV were not detected, continued surveillance remains important to ensure early detection and eradication if these viruses are introduced. The detection of multiple BSV species and co-infections further highlights the importance of sustained monitoring. Taken together, our findings demonstrate that banana viruses are widespread in Malawi, with prevalence shaped by genotype, informal seed practices, and regional planting dynamics. Strengthened phytosanitary measures, combined with the conservation and characterization of unique local germplasm, will be key to managing banana viruses and safeguarding production in Malawi.

## Supporting information

S1 Fig Prevalence of genotypes of banana mats that were sampled. This figure shows proportion of each detected banana genotype (AA, AAA, AAB and ABB) and unknown (not yet genotyped) banana surveys samples in this study.
(DOCX)

S2 Fig Types of banana virus infections detected in different banana cultivation sites and Zones of Malawi – created by Masangwa et al. Yellow stars stand for single virus detection in sample collected at that survey point. Red stars stand for the detection of two banana viruses in a single sample collected at that particular location on the map. Blue stars stand for three virus detection in a single sample. Round blue dots stand for four viruses detected in a single sample.
(DOCX)

S3 Fig Phylogenetic tree of BSV partial RNase H sequences. BSV Maximum Likelihood method phylogenetic tree constructed using Maximum Likelihood method and Jukes-Cantor model in MEGA from partial RNase H Gene sequences from this study (in red) and reference sequences from nt database (Genbank – NCBI) in black for Clade 1; blue for Clade 2 and green for Clade 3.
(DOCX)

S4 Fig Phylogenetic tree of BanMMV partial Coat protein sequences. BanMMV maximum Likelihood method phylogenetic tree was constructed using Jukes-Cantor model in MEGA from partial Coat protein (CP) from this study (in red) and reference sequences from nt database (Genbank – NCBI) in black.
(DOCX)

S1 Table Primers and PCR protocols used in this study. The name of targeted virus, name of primer, primer sequences, the amplicon size in base pairs (bp), annealing temperatures in degrees celcius (^o^C) and the source of each primer.
(DOCX)

S2 Table.
**(A) Kolmogorov-Smirnov and Shapiro-Wilk normality tests for banana cultivation systems across Malawi’s cultivation zones.** The columns of the table represent banana production system, statistic, degree of freedom (df) and significant (p-value) for Kolmogorov-Smirnov and Shapiro-Wilk. (**B) Kolmogorov-Smirnov and Shapiro-Wilk normality tests for different sources of banana mat in Malawi.** The columns of the table banana source of mat, statistic, degree of freedom (df) and significant (p-value) for Kolmogorov-Smirnov and Shapiro-Wilk. (**C) Kolmogorov-Smirnov and Shapiro-Wilk normality tests for different banana genotypes present in Malawi.** The columns of the table represent banana genotype Statistic, degree of freedom (df) and significant (p-value) for Kolmogorov-Smirnov and Shapiro-Wilk. (**D) Table. Kolmogorov-Smirnov and Shapiro-Wilk normality tests of different ages of banana mats in Malawi.** The columns of the table represent age of banana mat, Statistic, degree of freedom (df) and significant (p-value) for Kolmogorov-Smirnov and Shapiro-Wilk.
(DOCX)

S3 Table.
**(A)** Snedecor and Cochran’s normality test for banana viruses in banana cultivation zones. This S3A table columns correspond to the number of samples (n), the expected number of successes (np) and the expected number of failures (nq). (**B) Snedecor and Cochran’s normality test for banana viruses and age of banana mats.** This S3B table columns correspond to the number of samples (n), the expected number of successes (np) and the expected number of failures (nq). (**C) Snedecor and Cochran’s normality test for banana viruses under banana cultivation systems.** This S3C table columns correspond to the banana cultivation system number of samples (n), the expected number of successes (np) and the expected number of failures (nq). (**D) Snedecor and Cochran’s normality test for banana viruses infecting banana genotypes**. This S3D table columns correspond to the genotypes number of samples (n), the expected number of successes (np) and the expected number of failures (nq). (**E) Snedecor and Cochran’s normality test for banana viruses infecting banana source of mat.** This S3E table columns correspond to the source of mat number of samples (n), the expected number of successes (np) and the expected number of failures (nq).
(DOCX)

S4 Table Banana virus reference accessions from NCBI GenBank used to construct phylogenetic trees. The name of viruses (BBTV, BanMMV and BSV), the NCBI accession number, segment analyzed, origin of the accession and BSV species.
(DOCX)

S5 Table Survey data and IC-(RT)-PCR test results. This S5 Table, columns represent location, banana cultivation zone, district, age of sampled banana mat, the source (origin) of the mat, its name, genotype, field survey sample ID and IC- (RT)-PCR test results for BanMMV, BSV and BBTV. Red coloured genotypes: not characterized in this study, information derived from literature.
(XLSX)

S6 Table SSR results table showing clades for the sampled Malawi’s accessions. This S6 Table has accession’s local name, its related accession(s) in the banana core set collection and the Clade in which it is.
(DOCX)

S7 Table Association between banana cultivation zones and ages of banana mat in Malawi (Chi squared test). The columns of the S7 Table represent banana cultivation zones, and age of banana mats (1–3 yrs, 4–6 yrs and Over 6 yrs), total number of banana mat sampled, Chi-square value, degrees of freedom, p value and phi value.
(DOCX)

S8 Table Association between banana cultivation zones and cropping systems (Chi squared test). The columns of the S8 Table represent banana cultivation zones, banana cropping system (mono cropping and mixed cropping), total number of banana mat sampled per system, Chi-square value, degrees of freedom, p value and phi value.
(DOCX)

S9 Table Association between banana cultivation zones and banana genotypes (Chi squared test). The columns of the S9 Table represent banana cultivation zones, total number of banana mat sampled per each cultivation zone, banana genotypes (AAA, AAB and ABB), Chi-square value, degrees of freedom, p value and phi value.
(DOCX)

S10 Table Distribution of banana genotypes across four zones and infection prevalence of BBTV, BanMMV, and BSV species. The columns of the S10 Table represent banana cultivation zones, banana genotypes, number of samples, percentage (number) of virus infected mats per genotype.
(DOCX)

S11 Table Association between banana cultivation zones and sources of banana mat (Chi squared test). The columns of the S11 Table represent banana cultivation zones, source of banana mat (sharing, purchase and own-field), total number of banana mat sampled per each cultivation zone and source of mat, Chi-square value, degrees of freedom, p value and phi value.
(DOCX)

S12 Table Association between banana mat ages and source of mat (Chi squared test). The columns of the S12 Table represent age of banana mats, source of banana mats (sharing, purchase and own-field), total number of mat per each mat age category, Chi-square value, degrees of freedom, p value and phi value.
(DOCX)

S13 Table Association between cropping system and banana genotypes (Chi squared test). The columns of the S13 Table represent banana cultivation system, genotype (AAA, AAB and ABB), total number of mat per each cultivation system, Chi-square value, degrees of freedom, p value and phi value.
(DOCX)

S14 Table Association between banana mat ages and banana cultivation systems (Chi squared test). The columns of the S14 Table represent banana cultivation system, age of banana mat (1–3 yrs, 4–6 yrs and Over 6 yrs), total number of mat per each cultivation system, Chi-square value, degrees of freedom, p value and phi value.
(DOCX)

S15 Table Association between banana cropping systems and source of banana mats (Chi squared test). The columns of the S15 Table represent banana production system, source of banana mats (sharing, purchase and own-filed), total number of mat per each cultivation system, Chi-square value, degrees of freedom, p value and phi value.
(DOCX)

S16 Table Association between banana mat ages and banana genotypes (Chi squared test). The columns of the S16 Table represent age of banana mats, genotype (AAA, AAB, ABB), total number of mat per each cultivation system, Chi-square value, degrees of freedom, p value and phi value.
(DOCX)

S17 Table Association between banana mat source and banana genotypes (Chi squared test). The columns of the S17 Table represent source of banana mats, genotype (AAA, AAB and ABB), total number of mat per each cultivation system, Chi-square value, degrees of freedom, p value and phi value.
(DOCX)

S18 Table Virus detection by immunocapture-(RT)-PCR results. This S18 Table shows results of the IC-RT-PCR test results for BanMMV, BBTV, CMV, BBrMV and BSV species (BSMYV, BSOLV, BSIMV, BSVLac, BSGFV and BSCaV). Red color indicates IC-RT-PCR test positive results.
(XLSX)

S19 Table Percentage (numbers) of detected BSV species per cultivation Zone. This S19 Table percentage (numbers) of detected BSV species (BSCAV, BSGFV, BSLAC, BSIMV, BSMYV and BSOLV) in percent (number) per banana cultivation zones of Malawi.
(DOCX)

S20 Table Effect of age of banana mats on prevalences of banana viruses: BBTV, BanMMV and BSVs. Columns represent age of mat, number of mat, virus, prevalence (number), 95% CI = 95% confidence interval. *: significantly different (P < 0.05); ** very significantly different (P < 0.01); *** very highly significantly different (P < 0.001) and OR = odd number.
(DOCX)

S21 Table Effect of banana cultivation systems on prevalences of banana viruses: BBTV, BanMMV and BSVs. Columns represent age of mat, number of mat, virus, prevalence (number), 95% CI = 95% confidence interval. *: significantly different (P < 0.05); ** very significantly different (P < 0.01); *** very highly significantly different (P < 0.001) and and OR = odd number.
(DOCX)

S22 Table Banana virus isolates from this study and their NCBI GenBank accession numbers. Sequences generated from detected banana viruses in this study, segment analyzed and the assigned NCBI GenBank accession number.
(DOCX)
