# Supplementary material for: Surveillance and molecular characterization of banana viruses associated with Musa germplasm in Malawi
Source: PLoS One. 2026 Jan 29;21(1):e0306671. doi: 10.1371/journal.pone.0306671 (PMC12854425; doi:10.1371/journal.pone.0306671)
Supplement: S1 Fig — This figure shows proportion of each detected banana genotype (AA, AAA, AAB and ABB) and unknown (not yet genotyped) banana surveys samples in this study. (DOCX) [file pone.0306671.s001.docx]

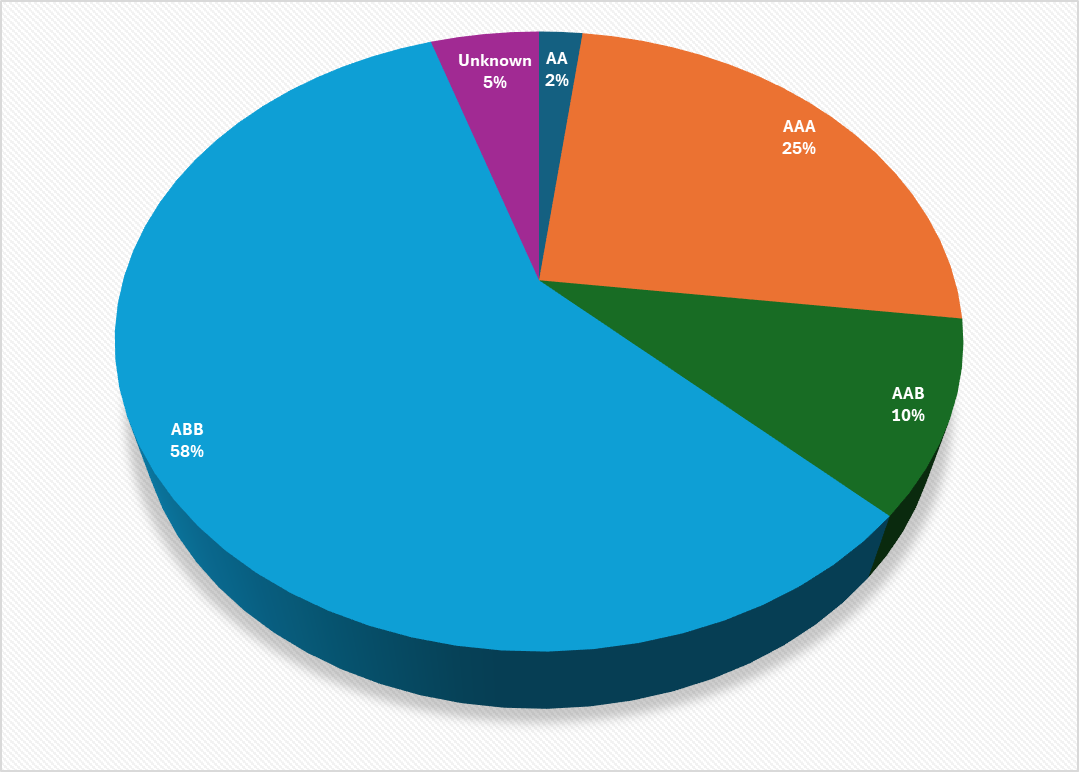


**S1 Fig. Prevalence of genotypes of banana mats that were sampled.** This figure shows proportion of each detected banana genotype (AA, AAA, AAB and ABB) and unknown (not yet genotyped) banana surveys samples in this study.
