# Supplementary material for: Surveillance and molecular characterization of banana viruses associated with Musa germplasm in Malawi
Source: PLoS One. 2026 Jan 29;21(1):e0306671. doi: 10.1371/journal.pone.0306671 (PMC12854425; doi:10.1371/journal.pone.0306671)
Supplement: S2 Fig — Yellow stars stand for single virus detection in sample collected at that survey point. Red stars stand for the detection of two banana viruses in a single sample collected at that particular location on the map. Blue stars stand for three virus detection in a single sample. Round blue dots stand for four viruses detected in a single sample. (DOCX) [file pone.0306671.s002.docx]

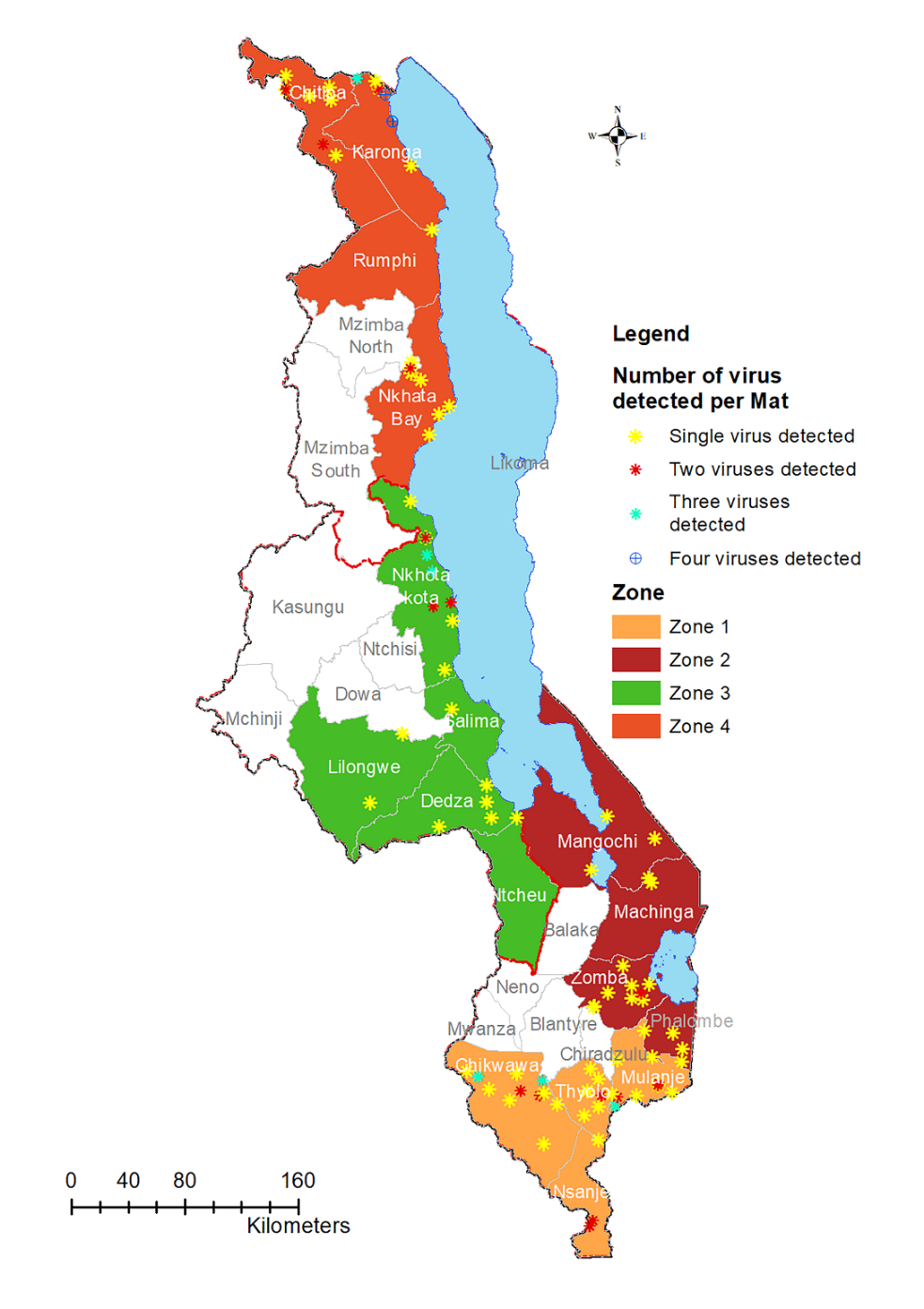


**S2 Fig. Types of banana virus infections detected in different banana cultivation sites and zones of Malawi - created by Masangwa et al.** Yellow stars stand for single virus detection in sample collected at that survey point. Red stars stand for the two banana viruses detection in a single sample collected at that particular point on the map. Blue stars stand for three virus detection in a single sample. Round blue dots stand for four viruses detected in a single sample.
