# Supplementary material for: Surveillance and molecular characterization of banana viruses associated with Musa germplasm in Malawi
Source: PLoS One. 2026 Jan 29;21(1):e0306671. doi: 10.1371/journal.pone.0306671 (PMC12854425; doi:10.1371/journal.pone.0306671)
Supplement: S3 Fig — BSV Maximum Likelihood method phylogenetic tree constructed using Maximum Likelihood method and Jukes-Cantor model in MEGA from partial RNase H Gene sequences from this study (in red) and reference sequences from nt database (Genbank – NCBI) in black for Clade 1; blue for Clade 2 and green for Clade 3. (DOCX) [file pone.0306671.s003.docx]

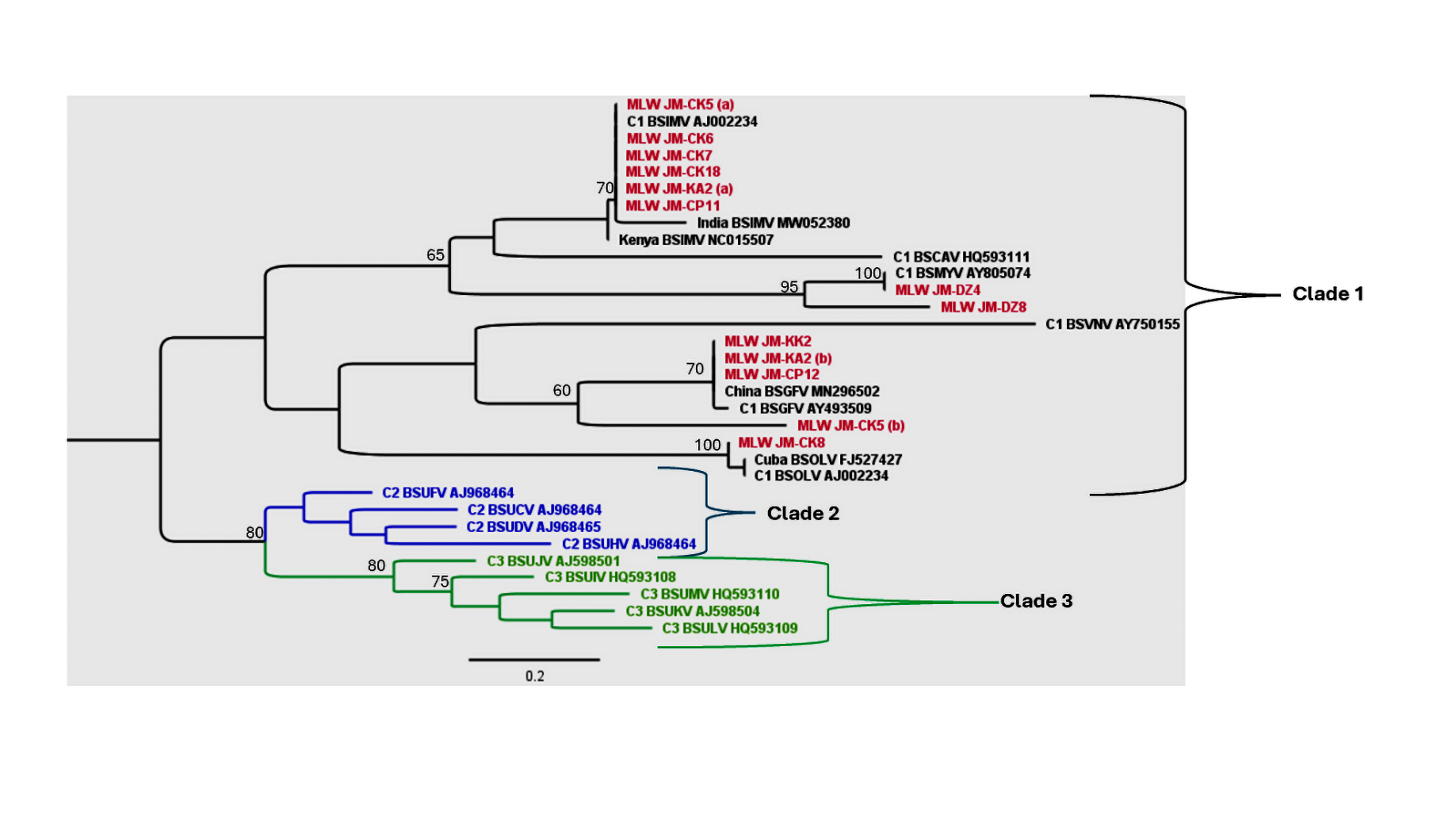


**S3 Fig. Phylogenetic tree of BSV partial RNase H sequences.** BSV Maximum Likelihood method phylogenetic tree constructed using Maximum Likelihood method and Jukes-Cantor model in MEGA from partial RNase H Gene sequences from this study (in red) and reference sequences from nt database (Genbank – NCBI) in black for Clade 1; blue for Clade 2 and green for Clade 3.
