# Supplementary material for: Surveillance and molecular characterization of banana viruses associated with Musa germplasm in Malawi
Source: PLoS One. 2026 Jan 29;21(1):e0306671. doi: 10.1371/journal.pone.0306671 (PMC12854425; doi:10.1371/journal.pone.0306671)
Supplement: S4 Fig — BanMMV maximum Likelihood method phylogenetic tree was constructed using Jukes-Cantor model in MEGA from partial Coat protein (CP) from this study (in red) and reference sequences from nt database (Genbank – NCBI) in black. (DOCX) [file pone.0306671.s004.docx]

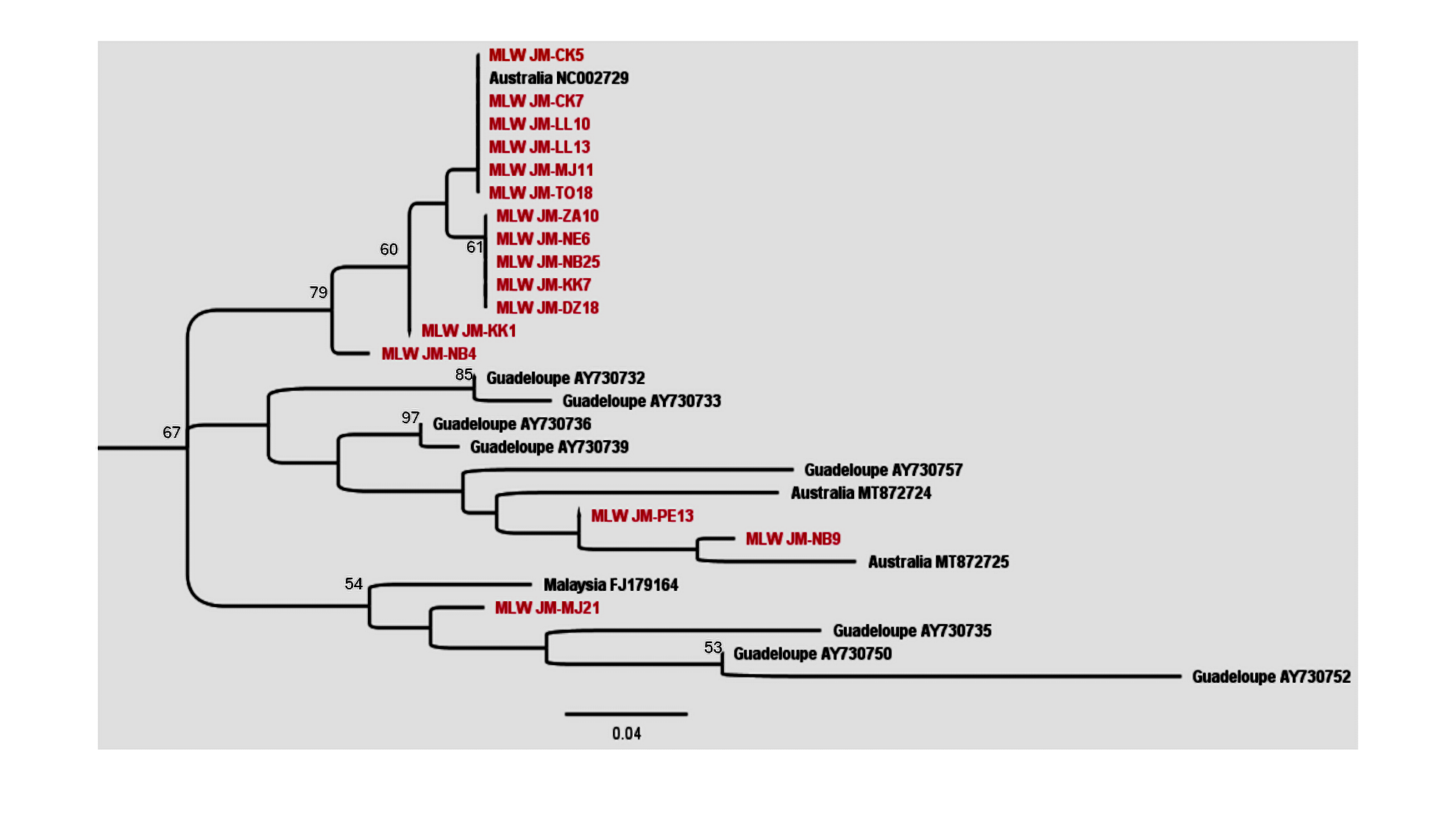


**S4 Fig. Phylogenetic tree of BanMMV partial Coat protein sequences.** BanMMV maximum Likelihood method phylogenetic tree constructed using Jukes-Cantor model in MEGA from partial Coat protein (CP) from this study (in red) and reference sequences from nt database (Genbank – NCBI) in black.
