# Supplementary material for: Surveillance and molecular characterization of banana viruses associated with Musa germplasm in Malawi
Source: PLoS One. 2026 Jan 29;21(1):e0306671. doi: 10.1371/journal.pone.0306671 (PMC12854425; doi:10.1371/journal.pone.0306671)
Supplement: S1 Table — The name of targeted virus, name of primer, primer sequences, the amplicon size in base pairs (bp), annealing temperatures in degrees celcius (oC) and the source of each primer. (DOCX) [file pone.0306671.s005.docx]

**S1 Table. Primers and PCR protocols used in this study.** The name of targeted virus, name of primer, primer sequences, the amplicon size in base pairs (bp), annealing temperatures in degrees celcius (^o^C) and the source of each primer.

| **Virus target** | **Primer Name** | **Primer F (5'-3')** | **Primer R (5'-3')** | **Amplicon size (bp)** | **Annealing temp. (oC)** | **Reference** |
| --- | --- | --- | --- | --- | --- | --- |
| CMV | CMV3’ | TTTTAGCCGTAAGCTGGATGGACAACCC | TATGATAAGAAGCTTGTTTCGCGCA | 500 | 60 | Bariana et al. [71]. |
| BBrMV | Bract | GACATCACCAAATTTGAATGGCACATGG | CCATTATCACTCGATCAATACCTCACAG | 650 and/or 700 | 60 | Rodoni et al [72] |
| BBTV | BBT | CTC GTC ATG TGC AAG GTT ATG TCG | GAA GTT CTC CAG CTA TTC ATTC GCC | 350 | 60 | Thomas and Dietzgen [73] |
| BanMMV | Poty 1- BanMMCP2 | GGATCCCGGGTTTTTTTTTTTTTTTTTV | TGCCAACTGAYGARGAGCTRAATGC | 280 | 60 | M. Sharman (pers. Communication) [74]  Gibbs and Mackenzie [76] |
| BanMMV | Poty-1 - BanMMCP9 | GGATCCCGGGTTTTTTTTTTTTTTTTTV | TCT GAT KCA GCA ATH ATS CC | 320 | 60 | M. Hanafi et al. [75] |
| BSOLV | RD | ATC TGA AGG TGT GTT GAT CAA TGC | GCTCACTCCGCATCTTATCAGTC | 522 | 60 | Geering, et al. [77] |
| BSGFV | GF | ACG AAC TAT CAC GAC TTG TTC AAG C | TCG GTG GAA TAG TCC TGA GTC TTC | 476 | 60 | Geering, et al. [77] |
| BSMYV | Mys | TAA AAG CAC AGC TCA GAA CAA ACC | CTC CGT GAT TTC GTG GTC | 589 | 60 | Geering, et al. [77] |
| BSCAV | Cav | AGG ATT GGA TGT GAA GTT TGA GC | ACC AAT AAT GCA AGG GAC GC | 782 | 60 | A. Geering (pers. communication) [78] |
| BSLACV | Lac | AAA AGC ACC GAA CAC AAG CC | CAT TGC TTA CGA CCT TCT CCAC | 700 | 60 | A. Geering (pers. communication) [78] |
| BSIMV | IM | TGC CAA CGA ATA CTA CAT CAA C | CAC CCA GAC TTT TCT TTC TAG C | 400 | 60 | Geering et al. [79] |
