# Supplementary material for: Surveillance and molecular characterization of banana viruses associated with Musa germplasm in Malawi
Source: PLoS One. 2026 Jan 29;21(1):e0306671. doi: 10.1371/journal.pone.0306671 (PMC12854425; doi:10.1371/journal.pone.0306671)
Supplement: S2 Table. — (A) Kolmogorov-Smirnov and Shapiro-Wilk normality tests for banana cultivation systems across Malawi’s cultivation zones. The columns of the table represent banana production system, statistic, degree of freedom (df) and significant (p-value) for Kolmogorov-Smirnov and Shapiro-Wilk. (B) Kolmogorov-Smirnov and Shapiro-Wilk normality tests for different sources of banana mat in Malawi. The columns of the table banana source of mat, statistic, degree of freedom (df) and significant (p-value) for Kolmogorov-Smirnov and Shapiro-Wilk. (C) Kolmogorov-Smirnov and Shapiro-Wilk normality tests for different banana genotypes present in Malawi. The columns of the table represent banana genotype Statistic, degree of freedom (df) and significant (p-value) for Kolmogorov-Smirnov and Shapiro-Wilk. (D) Table. Kolmogorov-Smirnov and Shapiro-Wilk normality tests of different ages of banana mats in Malawi. The columns of the table represent age of banana mat, Statistic, degree of freedom (df) and significant (p-value) for Kolmogorov-Smirnov and Shapiro-Wilk. (DOCX) [file pone.0306671.s006.docx]

**S2A Table. Kolmogorov–Smirnov and Shapiro–Wilk normality tests for banana cultivation systems** **across Malawi’s cultivation zones.** The columns of the table represent banana cultivation zones, production system, statistic, degree of freedom (df) and significant (p-value) for Kolmogorov–Smirnov and Shapiro–Wilk.

|  | Banana production system | Kolmogorov-Smirnov^a^ | | | Shapiro-Wilk | | |
| --- | --- | --- | --- | --- | --- | --- | --- |
|  |  | Statistic | df | Sig. | Statistic | df | Sig. |
| Zones of banana cultivation | Mono cropping | .151 | 4 | . | 0.993 | 4 | 0.972 |
|  | Mixed cropping | .151 | 4 | . | 0.993 | 4 | 0.972 |
| a. Lilliefors Significance Correction | | | |  |  |  |  |

**S2B Table. Kolmogorov-Smirnov and Shapiro-Wilk normality tests for different sources of banana mat present in Malawi.** The columns of the table represent banana source of mat, statistic, degree of freedom (df) and significant (p-value) for Kolmogorov-Smirnov and Shapiro-Wilk.

|  | Mat_source | Kolmogorov-Smirnov^a^ | | | Shapiro-Wilk | | |
| --- | --- | --- | --- | --- | --- | --- | --- |
|  |  | Statistic | df | Sig. | Statistic | df | Sig. |
| Mat_number_per_source | Floods | 0.192 | 4 | . | 0.971 | 4 | 0.850 |
|  | Government | 0.162 | 4 | . | 0.989 | 4 | 0.952 |
|  | Own-farm | 0.269 | 4 | . | 0.878 | 4 | 0.332 |
|  | Purchase | 0.260 | 4 | . | 0.827 | 4 | 0.161 |
|  | Relatives | 0.285 | 4 | . | 0.899 | 4 | 0.427 |
|  | NGO | 0.385 | 3 | . | 0.750 | 3 | 0.000 |
| Percent_mat_per_source | Floods | 0.210 | 4 | . | 0.982 | 4 | 0.911 |
|  | Government | 0.203 | 4 | . | 0.980 | 4 | 0.899 |
|  | Own-farm | 0.293 | 4 | . | 0.918 | 4 | 0.528 |
|  | Purchase | 0.151 | 4 | . | 0.993 | 4 | 0.972 |
|  | Relatives | 0.267 | 4 | . | 0.904 | 4 | 0.449 |
|  | NGO | 0.385 | 3 | . | 0.750 | 3 | 0.000 |
| a. Lilliefors Significance Correction | | | | | |  |  |

**S2C Table. Kolmogorov-Smirnov and Shapiro-Wilk normality tests for different banana genotypes present in Malawi.** The columns of the table represent banana genotype, statistic, degree of freedom (df) and significant (p-value) for Kolmogorov-Smirnov and Shapiro-Wilk.

|  | Banana_genotype | Kolmogorov-Smirnov^a^ | | | Shapiro-Wilk | | |
| --- | --- | --- | --- | --- | --- | --- | --- |
|  |  | Statistic | df | Sig. | Statistic | df | Sig. |
| All_genotypes | AA | .192 | 4 | . | .971 | 4 | .850 |
|  | AAA | .222 | 4 | . | .970 | 4 | .842 |
|  | AAB | .237 | 4 | . | .939 | 4 | .650 |
|  | ABB | .202 | 4 | . | .969 | 4 | .836 |
|  | Unknown | .302 | 4 | . | .827 | 4 | .161 |
| a. Lilliefors Significance Correction | | |  |  |  |  |  |

**Table S2D. Kolmogorov-Smirnov and Shapiro-Wilk normality tests of different ages of banana mats in Malawi**. The columns of the table represent age of banana mat, statistic, degree of freedom (df) and significant (p-value) for Kolmogorov-Smirnov and Shapiro-Wilk.

| . | Age_of_banana_mat | Kolmogorov-Smirnov^a^ | | | Shapiro-Wilk | | |
| --- | --- | --- | --- | --- | --- | --- | --- |
|  |  | Statistic | df | Sig. | Statistic | df | Sig. |
| Percent_mat_ages | 1-3yrs | .242 | 4 | . | .874 | 4 | .312 |
|  | 4-6yrs | .215 | 4 | . | .937 | 4 | .639 |
|  | Over_6yrs | .250 | 4 | . | .963 | 4 | .797 |
| Ages_of_all_matsLog | 1-3yrs | .316 | 4 | . | .816 | 4 | .134 |
|  | 4-6yrs | .280 | 4 | . | .848 | 4 | .218 |
|  | Over_6yrs | .212 | 4 | . | .933 | 4 | .611 |
| a. Lilliefors Significance Correction | | | | | |  |  |
