# Supplementary material for: Surveillance and molecular characterization of banana viruses associated with Musa germplasm in Malawi
Source: PLoS One. 2026 Jan 29;21(1):e0306671. doi: 10.1371/journal.pone.0306671 (PMC12854425; doi:10.1371/journal.pone.0306671)
Supplement: S3 Table. — (A) Snedecor and Cochran’s normality test for banana viruses in banana cultivation zones. This S3A table columns correspond to the number of samples (n), the expected number of successes (np) and the expected number of failures (nq). (B) Snedecor and Cochran’s normality test for banana viruses and age of banana mats. This S3B table columns correspond to the number of samples (n), the expected number of successes (np) and the expected number of failures (nq). (C) Snedecor and Cochran’s normality test for banana viruses under banana cultivation systems. This S3C table columns correspond to the banana cultivation system number of samples (n), the expected number of successes (np) and the expected number of failures (nq). (D) Snedecor and Cochran’s normality test for banana viruses infecting banana genotypes. This S3D table columns correspond to the genotypes number of samples (n), the expected number of successes (np) and the expected number of failures (nq). (E) Snedecor and Cochran’s normality test for banana viruses infecting banana source of mat. This S3E table columns correspond to the source of mat number of samples (n), the expected number of successes (np) and the expected number of failures (nq). (DOCX) [file pone.0306671.s007.docx]

**S3A Table. Snedecor and Cochran’s normality test for banana viruses in banana cultivation zones.** This S3A Table has the number of samples (n), the expected number of successes (np) and the expected number of failures (nq).

| **Cultivation zones** | **BBTV** | **BanMMV** | **BSV** |
| --- | --- | --- | --- |
| n | 275 | 275 | 275 |
| np | 29 | 35 | 62 |
| nq | 246 | 240 | 213 |

**S3B Table. Snedecor and Cochran’s normality test for banana viruses and age of banana mats.** This S3B Table has the number of samples (n), the expected number of successes (np) and the expected number of failures (nq).

| **Age** | **BBTV** | | **BanMMV** | | **BSV** | |
| --- | --- | --- | --- | --- | --- | --- |
| n | 275 | | 275 | | 275 | |
| np | 29 | | 35 | | 62 | |
| nq | 246 | | 240 | | 213 | |
|  |  |  | |  | |  |

**S3C Table. Snedecor and Cochran’s normality test for banana viruses under banana cultivation systems.** This S3C Table has the banana cultivation system number of samples (n), the expected number of successes (np) and the expected number of failures (nq).

| **Cultivation system** | **BBTV** | **BanMMV** | | | **BSV** |  |
| --- | --- | --- | --- | --- | --- | --- |
| n | 275 | | 275 | 275 | | |
| np | 29 | | 35 | 62 | | |
| nq | 246 | | 240 | 213 | | |

**S3D Table. Snedecor and Cochran’s normality test for banana viruses infecting banana genotypes.** This S3D Table has the genotypes number of samples (n), the expected number of successes (np) and the expected number of failures (nq).

| **Genotypes** | **BBTV** | | **BanMMV** | **BSV** | | |
| --- | --- | --- | --- | --- | --- | --- |
| n | 256 | | 256 | 256 | | |
| np | 26 | | 35 | 58 | | |
| nq | | 230 | 221 | | 198 |  |

**S3E Table. Snedecor and Cochran’s normality test for banana viruses infecting banana source of mat.** This S3E Table has the source of mat number of samples (n), the expected number of successes (np) and the expected number of failures (nq).

| **Source of mat** | **BBTV** | **BanMMV** | **BSV** |
| --- | --- | --- | --- |
| n | 254 | 254 | 254 |
| np | 24 | 32 | 56 |
| nq | 230 | 222 | 198 |
