# Supplementary material for: Surveillance and molecular characterization of banana viruses associated with Musa germplasm in Malawi
Source: PLoS One. 2026 Jan 29;21(1):e0306671. doi: 10.1371/journal.pone.0306671 (PMC12854425; doi:10.1371/journal.pone.0306671)
Supplement: S4 Table — The name of viruses (BBTV, BanMMV and BSV), the NCBI accession number, segment analyzed, origin of the accession and BSV species. (DOCX) [file pone.0306671.s008.docx]

**S4 Table.** **Banana virus reference accessions from NCBI GenBank used to construct phylogenetic trees.** The name of viruses (BBTV, BanMMV and BSV), the NCBI accession number, segment analyzed, origin of the accession and BSV species.

| **Accession number** | **Segment analyzed** | **Origin** | **Accession number** | **Segment analyzed** | **Origin** | **Accession number** | **Segment analyzed** | **BSV species** | **Origin** |
| --- | --- | --- | --- | --- | --- | --- | --- | --- | --- |
| AY450396 | DNA-R | China | NC002729 | Coat | Australia | AY493509 | Ribonuclease H | BSGFV | Ecuador |
| AB250954 | DNA-R | Philippines | AF314662 | Coat | Australia | MN296502 | Ribonuclease H | BSGFV | China |
| AB108454 | DNA-R | Japan | AY730732 | Coat | Guadeloupe | AJ002234 | Ribonuclease H | BSOLV | Unknown |
| KM607594 | DNA-R | Philippines | AY730733 | Coat | Guadeloupe | FJ527427 | Ribonuclease H | BSOLV | Cuba |
| MN017713 | DNA-R | Indonesia | AY730736 | Coat | Guadeloupe | HQ593111 | Ribonuclease H | BSCAV | Kenya |
| KM607660 | DNA-R | USA | MT872725 | Coat | Papua New Guinea | HQ593107 | Ribonuclease H | BSUAV | Uganda |
| KM607599 | DNA-R | USA | MT872724 | Coat | Papua New Guinea | HQ659760 | Ribonuclease H | BSIMV | Australia |
| KM607673 | DNA-R | Samoa | AY730757 | Coat | Guadeloupe | NC015507 | Ribonuclease H | BSIMV | Kenya |
| KM607672 | DNA-R | Samoa | FJ179164 | Coat | Malaysia | MW052380 | Ribonuclease H | BSIMV | India |
| AF416465 | DNA-R | Egypt | AY730750 | Coat | Guadeloupe | AY805074 | Ribonuclease H | BSMYV | Australia |
| KX868958 | DNA-R | India | AY730752 | Coat | Guadeloupe | AY750155 | Ribonuclease H | BSVNV | Vietnam |
| JQ820453 | DNA-R | Malawi |  |  |  | HQ593108 | Ribonuclease H | BSUIV | Uganda |
| ON934241 | DNA-R | Malawi |  |  |  | HQ593110 | Ribonuclease H | BSUMV | Uganda |
| MH795415 | DNA-R | Tanzania |  |  |  | HQ593109 | Ribonuclease H | BSULV | Uganda |
| JQ820459 | DNA-R | Rwanda |  |  |  |  |  |  |  |
| JF957634 | DNA-R | Tonga |  |  |  |  |  |  |  |
| KY322773 | DNA-R | USA |  |  |  |  |  |  |  |
