# Supplementary material for: Surveillance and molecular characterization of banana viruses associated with Musa germplasm in Malawi
Source: PLoS One. 2026 Jan 29;21(1):e0306671. doi: 10.1371/journal.pone.0306671 (PMC12854425; doi:10.1371/journal.pone.0306671)
Supplement: S6 Table — This S6 Table has accession’s local name, its related accession(s) in the banana core set collection and the Clade in which it is. (DOCX) [file pone.0306671.s010.docx]

**S6 Table. SSR results table showing clades for the sampled Malawi’s accessions.** This S6 Table has accession’s local name, its related accession(s) in the bananan core set collection and the Clade in which it is.

| **ACCESSION'S NAME** | **RELATED ACCESSION(S)**  **IN BANANA CORE SET COLLECTION** (Christelová et al. [46]). | **CLADE** |
| --- | --- | --- |
| Kabuthu | the same SSR profile as the Mtwishe ITC 1540, and Dwarf Cavendish ITC1720 | Cavendish clade |
| Kaluma |  |  |
| Kalaghasya |  |  |
| Kabuthu wamkulu |  |  |
| Kafupi NB |  |  |
| Kafupi KA |  |  |
| Khazanga-Chididi |  |  |
| Mulanje |  |  |
| Mthwika |  | Mchare clade |
| Suweshi |  |  |
| Muli | closely related to Leite ITC0277 | Rio clade |
| Matoki |  | Red clade |
| Zomba red |  |  |
| Mwamnyira Green |  |  |
| Zomba green |  |  |
| Nasili | same SSR profile as ITC1543; ITC1555; ITC1556; ITC1753; ITC1356 |  |
| Chisili  Ngerezi |  | Lujugira/Mutika clade |
|  |  |  |
| Uganda BV | same SSR profile as ITC0082 ITC1557; ITC1630; ITC1760; ITC0081 |  |
| Uganda KA |  |  |
| Ndifu |  |  |
| Munowa |  |  |
| Kapeni |  |  |
| Khombolimbo | same SSR profile as ITC0033; ITC0631 | Plantain clade |
| Khazanga wamngono |  |  |
| Kazanga Wamkulu | same SSR profile as ITC0114 |  |
| Matoki |  |  |
| Zeru |  |  |
| Ingegho |  |  |
| Mbilindola |  | Monthan clade |
| Mabere |  |  |
| Sukali |  | Silk clade |
| Zanda |  | Pisang Awak clade |
