# Supplementary material for: Surveillance and molecular characterization of banana viruses associated with Musa germplasm in Malawi
Source: PLoS One. 2026 Jan 29;21(1):e0306671. doi: 10.1371/journal.pone.0306671 (PMC12854425; doi:10.1371/journal.pone.0306671)
Supplement: S7 Table — The columns of the S7 Table represent banana cultivation zones, and age of banana mats (1–3 yrs, 4–6 yrs and Over 6 yrs), total number of banana mat sampled, Chi-square value, degrees of freedom, p value and phi value. (DOCX) [file pone.0306671.s011.docx]

**S7 Table. Association between banana cultivation zones and ages of banana mat in Malawi (Chi squared test).** The columns of the S7 Table are: banana cultivation zones, and age of banana mats (1-3 yrs, 4-6 yrs and Over 6 yrs), Total, Chi-square value, degrees of freedom, p value and phi value.

| Banana Cultivation Zones | Age of Banana mats | | | Total | χ² | df | p | Phi (φ) |
| --- | --- | --- | --- | --- | --- | --- | --- | --- |
|  | 1-3 yrs | 4-6 yrs | Over 6 yrs |  |  |  |  |  |
| Zone 1 | 57 % (40) | 3 % (2) | 40 % (28) | 100 % (70) |  |  |  |  |
| Zone 2 | 32 % (21) | 23 % (15) | 45 % (30) | 100 % (66) |  |  |  |  |
| Zone 3 | 39 % (25) | 12 % (8) | 49 % (32) | 100 % (65) |  |  |  |  |
| Zone 4 | 28 % (21) | 18 % (13) | 54 % (40) | 100 % (74) |  |  |  |  |
| Total | 39% (107) | 14 % (38) | 47 % (130) | 100 % (275) | 21.286 | 6 | 0.002 | 0.278 |
