# Supplementary material for: Surveillance and molecular characterization of banana viruses associated with Musa germplasm in Malawi
Source: PLoS One. 2026 Jan 29;21(1):e0306671. doi: 10.1371/journal.pone.0306671 (PMC12854425; doi:10.1371/journal.pone.0306671)
Supplement: S8 Table — The columns of the S8 Table represent banana cultivation zones, banana cropping system (mono cropping and mixed cropping), total number of banana mat sampled per system, Chi-square value, degrees of freedom, p value and phi value. (DOCX) [file pone.0306671.s012.docx]

**S8 Table. Association between banana cultivation zones and cropping systems (Chi squared test). T**he columns of the S8 Table are: banana cultivation zones, banana cropping system (mono cropping and mixed cropping), total number of banana mat sampled per system, Chi-square value, degrees of freedom, p value and phi value.

| Banana Cultivation zones | Banana cropping system | | Total | χ² | df | p | Phi (φ) |
| --- | --- | --- | --- | --- | --- | --- | --- |
|  | Mono cropping | Mixed cropping |  |  |  |  |  |
| Zone 1 | 26 % (18) | 74 % (52) | 100 % (70) |  |  |  |  |
| Zone 2 | 49 % (32) | 51 % (34) | 100 % (66) |  |  |  |  |
| Zone 3 | 57 % (37) | 43 % (28) | 100 % (65) |  |  |  |  |
| Zone 4 | 27 % (20) | 73 % (54) | 100 % (74) |  |  |  |  |
| Total | 39 % (107) | 61 % (168) | 100 % (275) | 20.942 | 3 | 0.000 | 0.276 |
