# Supplementary material for: Surveillance and molecular characterization of banana viruses associated with Musa germplasm in Malawi
Source: PLoS One. 2026 Jan 29;21(1):e0306671. doi: 10.1371/journal.pone.0306671 (PMC12854425; doi:10.1371/journal.pone.0306671)
Supplement: S9 Table — The columns of the S9 Table represent banana cultivation zones, total number of banana mat sampled per each cultivation zone, banana genotypes (AAA, AAB and ABB), Chi-square value, degrees of freedom, p value and phi value. (DOCX) [file pone.0306671.s013.docx]

**S9 Table. Association between banana cultivation zones and banana genotypes (Chi squared test).** The columns of the S9 Table are: banana cultivation zones, total number of banana mat sampled per each cultivation zone, banana genotypes (AAA, AAB and ABB), Chi-square value, degrees of freedom, p value and phi value.

| Banana Cultivation Zone | Genotype | | | Total | χ² | df | p | Phi (φ) |
| --- | --- | --- | --- | --- | --- | --- | --- | --- |
|  | AAA | AAB | ABB |  |  |  |  |  |
| Zone 1 | 36 % (24) | 8 % (5) | 56 % (37) | 100 % (66) |  |  |  |  |
| Zone 2 | 21 % (14) | 14 % (9) | 65 % (42) | 100 % (65) |  |  |  |  |
| Zone 3 | 15 % (9) | 6 % (4) | 79 % (48( | 100 % (61) |  |  |  |  |
| Zone 4 | 28 % (18) | 17 % (11) | 55 % (35) | 100 % (64) |  |  |  |  |
| Total | 25 % (64) | 11 % (29) | 63 % (162) | 100 % (256) | 14.363 | 6 | 0.026 | 0.237 |
