# Supplementary material for: Surveillance and molecular characterization of banana viruses associated with Musa germplasm in Malawi
Source: PLoS One. 2026 Jan 29;21(1):e0306671. doi: 10.1371/journal.pone.0306671 (PMC12854425; doi:10.1371/journal.pone.0306671)
Supplement: S10 Table — The columns of the S10 Table represent banana cultivation zones, banana genotypes, number of samples, percentage (number) of virus infected mats per genotype. (DOCX) [file pone.0306671.s014.docx]

**S10 Table. Distribution of banana genotypes across four zones and infection prevalence of BBTV, BanMMV, and BSV species**. The columns of the S10 Table represent banana cultivation zones, banana genotypes, number of samples, percentage (number) of virus infected mats per genotype

| Banana cultivation zones | Banana Genotypes | Number | Percentage (number) of virus infected mats per genotype in a zone | | |
| --- | --- | --- | --- | --- | --- |
|  |  |  | BBTV | BanMMV | BSV |
| Zone 1 | AA | 1% (1) | 0% (0) | 0% (0) | 0% (0) |
|  | AAA | 39% (27) | 23% (6) | 15% (4) | 4% (1) |
|  | AAB | 7% (5) | 40% (2) | 40% (2) | 20% (1) |
|  | AB | 0% (0) | - | - | - |
|  | ABB | 49% (34) | 3% (1) | 37% (14) | 47% (18) |
|  | Unknown | 4% (3) | 0% (0) | 0% (0) | 0% (0) |
| Zone 2 | AA | 0% 0 | - | - | - |
|  | AAA | 21% (14) | 36% (5) | 0% (0) | 7% (1) |
|  | AAB | 12% (8) | 0% (0) | 0% (0) | 38% (2) |
|  | AB | 0% (0) | - | - | - |
|  | ABB | 65% (43) | 2% (1) | 23% (10) | 9% (4) |
|  | Unknown | 2% (1) | 0% (0) | 0% (0) | 0% (0) |
| Zone 3 | AA | 3% (2) | 0% (0) | 0% (0) | 0% (0) |
|  | AAA | 14% (9) | 11% (1) | 11% (1) | 22% (2) |
|  | AAB | 6% (4) | 0 % (0) | 25% (1) | 0% (0) |
|  | AB | 0% (0) | - | - | - |
|  | ABB | 74% (48) | 9% (4) | 15% (7) | 15% (7) |
|  | Unknown | 3% (2) | 50% (1) | 50% (1) | 0% (0) |
| Zone 4 | AA | 3% (2) | 50% (1) | 0% (0) | 50% (1) |
|  | AAA | 23% (17) | 35% (6) | 24% (4) | 6% (1) |
|  | AAB | 15% (11) | 0% (0) | 20% (2) | 30% (3) |
|  | AB | 7% (5) | 0% (0) | 0% (0) | 40% (2) |
|  | ABB | 49% (36) | 3% (1) | 3% (1) | 41% (13) |
|  | Unknown | 4% (3) | 0% (0) | 0% (0) | 0% (0) |
