# Supplementary material for: Surveillance and molecular characterization of banana viruses associated with Musa germplasm in Malawi
Source: PLoS One. 2026 Jan 29;21(1):e0306671. doi: 10.1371/journal.pone.0306671 (PMC12854425; doi:10.1371/journal.pone.0306671)
Supplement: S11 Table — The columns of the S11 Table represent banana cultivation zones, source of banana mat (sharing, purchase and own-field), total number of banana mat sampled per each cultivation zone and source of mat, Chi-square value, degrees of freedom, p value and phi value. (DOCX) [file pone.0306671.s015.docx]

**S11 Table. Association between banana cultivation zones and sources of banana mat (Chi squared test).** The columns of the S11 Table represent banana cultivation zones, source of banana mat (sharing, purchase and own field), total number of banana mat sampled per each cultivation zone and source of mat, Chi-square value, degrees of freedom, p value and phi value.

| Banana Cultivation Zone | Source of banana mat | | | Total | χ² | df | p | Phi (φ) |
| --- | --- | --- | --- | --- | --- | --- | --- | --- |
|  | Sharing | Purchase | Own field |  |  |  |  |  |
| Zone 1 | 75 % (48) | 11 % (7) | 14 % (9) | 100 % (64) |  |  |  |  |
| Zone 2 | 82 % (49) | 10 % (6) | 8 % (5) | 100 % (60) |  |  |  |  |
| Zone 3 | 81 % (51) | 14 % (9) | 5 % (3) | 100 % (63) |  |  |  |  |
| Zone 4 | 67 % (45) | 13 % (9) | 19 % (13) | 100 % (67) |  |  |  |  |
| Total | 76 % (193) | 12 % (31) | 12 % (30) | 100 % (254) | 8.592 | 6 | 0.198 | 0.184 |
