# Supplementary material for: Surveillance and molecular characterization of banana viruses associated with Musa germplasm in Malawi
Source: PLoS One. 2026 Jan 29;21(1):e0306671. doi: 10.1371/journal.pone.0306671 (PMC12854425; doi:10.1371/journal.pone.0306671)
Supplement: S12 Table — The columns of the S12 Table represent age of banana mats, source of banana mats (sharing, purchase and own-field), total number of mat per each mat age category, Chi-square value, degrees of freedom, p value and phi value. (DOCX) [file pone.0306671.s016.docx]

**S12 Table. Association between banana mat ages and source of mat (Chi squared test).** The columns of the S12 Table represent age of banana mats, source of banana mats (sharing, purchase and own-field), total number of mat per each mat age category, Chi-square value, degrees of freedom, p value and phi value.

| Age of banana mats | Source of banana mats | | | Total | χ² | df | p | Phi (φ) |
| --- | --- | --- | --- | --- | --- | --- | --- | --- |
|  | Sharing | Purchase | Own field |  |  |  |  |  |
| 1-3 yrs | 62 % (59) | 24 % (23) | 14 % (13) | 100 % (106) |  |  |  |  |
| 4-6 yrs | 89 % (31) | 3 % (1) | 9 % (3) | 100 % (38) |  |  |  |  |
| Over 6 yrs | 83 % (103) | 6 % (7) | 11 % (14) | 100 % (128) |  |  |  |  |
| Total | 76 % (193) | 12 % (31) | 12 % (30) | 100 % (267) | 24.34 | 3 | 0.000 | 0.302 |
