# Supplementary material for: Surveillance and molecular characterization of banana viruses associated with Musa germplasm in Malawi
Source: PLoS One. 2026 Jan 29;21(1):e0306671. doi: 10.1371/journal.pone.0306671 (PMC12854425; doi:10.1371/journal.pone.0306671)
Supplement: S13 Table — The columns of the S13 Table represent banana cultivation system, genotype (AAA, AAB and ABB), total number of mat per each cultivation system, Chi-square value, degrees of freedom, p value and phi value. (DOCX) [file pone.0306671.s017.docx]

**S13 Table. Association between cropping system and banana genotypes (Chi squared test).** The columns of the S13 Table represent banana cultivation system, genotype (AAA, AAB and ABB), total number of mat per each cultivation system, Chi-square value, degrees of freedom, p value and phi value.

| Banana cultivation system | Genotype | | | Total | χ² | df | p | Phi (φ) |
| --- | --- | --- | --- | --- | --- | --- | --- | --- |
|  | AAA | AAB | ABB |  |  |  |  |  |
| Mono cropping | 18 % (17) | 9 % (9) | 73 % (71) | 100 % (97) |  |  |  |  |
| Mixed cropping | 30 % (48) | 13 % (20) | 57 % (91) | 100 % (159) |  |  |  |  |
| Total | 25 % (65) | 11 % (29) | 63 % (162) | 100 % (256) | 7.19 | 2 | 0.027 | 0.168 |
