# Supplementary material for: Surveillance and molecular characterization of banana viruses associated with Musa germplasm in Malawi
Source: PLoS One. 2026 Jan 29;21(1):e0306671. doi: 10.1371/journal.pone.0306671 (PMC12854425; doi:10.1371/journal.pone.0306671)
Supplement: S14 Table — The columns of the S14 Table represent banana cultivation system, age of banana mat (1–3 yrs, 4–6 yrs and Over 6 yrs), total number of mat per each cultivation system, Chi-square value, degrees of freedom, p value and phi value. (DOCX) [file pone.0306671.s018.docx]

**S14 Table. Association between banana mat ages and banana cultivation systems (Chi squared test).** The columns of the S14 Table represent banana cultivation system, age of banana mat (1-3 yrs, 4-6 yrs and Over 6 yrs), total number of mat per each cultivation system, Chi-square value, degrees of freedom, p value and phi value

| Banana cultivation system | Age of banana mat | | | Total | χ² | df | p | Phi (φ) |
| --- | --- | --- | --- | --- | --- | --- | --- | --- |
|  | 1-3 yrs | 4-6 yrs | Over 6 yrs |  |  |  |  |  |
| Mono cropping | 46 % (49) | 16 %  (17) | 38 %  (41) | 100 %  (107) |  |  |  |  |
| Mixed cropping | 35 % (58) | 12 %  (21) | 53 %  (89) | 100 %  (168) |  |  |  |  |
| Total | 39 %  (107) | 14 %  (38) | 47 %  (130) | 100 %  (275) | 5.648 | 2 | 0.059 | 0.143 |
