# Supplementary material for: Surveillance and molecular characterization of banana viruses associated with Musa germplasm in Malawi
Source: PLoS One. 2026 Jan 29;21(1):e0306671. doi: 10.1371/journal.pone.0306671 (PMC12854425; doi:10.1371/journal.pone.0306671)
Supplement: S15 Table — The columns of the S15 Table represent banana production system, source of banana mats (sharing, purchase and own-filed), total number of mat per each cultivation system, Chi-square value, degrees of freedom, p value and phi value. (DOCX) [file pone.0306671.s019.docx]

**S15 Table. Association between banana cropping systems and source of banana mats (Chi squared test).** The columns of the S15 Table represent banana production system, source of banana mats (sharing, purchase and own-filed), total number of mat per each cultivation system and genotype, Chi-square value, degrees of freedom, p value and phi value.

| Banana production System | Source of banana mats | | | Total | χ² | df | p | Phi (φ) |
| --- | --- | --- | --- | --- | --- | --- | --- | --- |
|  | Sharing | Purchase | Own-field |  |  |  |  |  |
| Mono cropping | 78 % (78) | 15 % (15) | 7 % (7) | 100 % (100) |  |  |  |  |
| Mixed cropping | 75 % (115) | 10 % (16) | 15 % (23) | 100 %  (154) |  |  |  |  |
| Total | 76 % (193) | 12 % (31) | 12 % (30) | 100 % (254) | 4.376 | 2 | 0.112 | 0.131 |
