# Supplementary material for: Surveillance and molecular characterization of banana viruses associated with Musa germplasm in Malawi
Source: PLoS One. 2026 Jan 29;21(1):e0306671. doi: 10.1371/journal.pone.0306671 (PMC12854425; doi:10.1371/journal.pone.0306671)
Supplement: S16 Table — The columns of the S16 Table represent age of banana mats, genotype (AAA, AAB, ABB), total number of mat per each cultivation system, Chi-square value, degrees of freedom, p value and phi value. (DOCX) [file pone.0306671.s020.docx]

**S16 Table. Association between banana mat ages and banana genotypes (Chi squared test).** The columns of the S16 Table represent age of banana mats, genotype (AAA, AAB, ABB), total number of mat per each cultivation system, Chi-square value, degrees of freedom, p value and phi value.

| Age of banana mats | Genotype | | | Total | χ² | df | p | Phi (φ) |
| --- | --- | --- | --- | --- | --- | --- | --- | --- |
|  | AAA | AAB | ABB |  |  |  |  |  |
| 1-3 yrs | 27% (26) | 16% (15) | 57% (55) | 96 |  |  |  |  |
| 4-6 yrs | 27% (10) | 8% (3) | 65% (24) | 37 |  |  |  |  |
| Over 6 yrs | 24% (29) | 9% (11) | 67% (83) | 123 |  |  |  |  |
| Total | 25 % (65) | 11% (29) | 63% (162) | 255 | 3.729 | 4 | 0.459 | 0.121 |
