# Supplementary material for: Surveillance and molecular characterization of banana viruses associated with Musa germplasm in Malawi
Source: PLoS One. 2026 Jan 29;21(1):e0306671. doi: 10.1371/journal.pone.0306671 (PMC12854425; doi:10.1371/journal.pone.0306671)
Supplement: S17 Table — The columns of the S17 Table represent source of banana mats, genotype (AAA, AAB and ABB), total number of mat per each cultivation system, Chi-square value, degrees of freedom, p value and phi value. (DOCX) [file pone.0306671.s021.docx]

**S17 Table. Association between banana mat source and banana genotypes (Chi squared test).** The columns of the S17 Table represent source of banana mats, genotype (AAA, AAB and ABB), total number of mat per each cultivation system, Chi-square value, degrees of freedom, p value and phi value.

| Source of banana mats | Genotype | | | Total | χ² | df | p | Phi (φ) |
| --- | --- | --- | --- | --- | --- | --- | --- | --- |
|  | AAA | AAB | ABB |  |  |  |  |  |
| Sharing | 67 % (39) | 81 % (22) | 78.4 % (120) | 181 |  |  |  |  |
| Purchase | 17 % (10) | 15 % (4) | 9.2 % (14) | 28 |  |  |  |  |
| Own farm | 16 % (9) | 3 % (1) | 12.4 % (19) | 29 |  |  |  |  |
| Total | 64 | 28 | 155 | 238 | 5.521 | 4 | 0.238 | 0.152 |
