# Supplementary material for: Surveillance and molecular characterization of banana viruses associated with Musa germplasm in Malawi
Source: PLoS One. 2026 Jan 29;21(1):e0306671. doi: 10.1371/journal.pone.0306671 (PMC12854425; doi:10.1371/journal.pone.0306671)
Supplement: S19 Table — This S19 Table percentage (numbers) of detected BSV species (BSCAV, BSGFV, BSLAC, BSIMV, BSMYV and BSOLV) in percent (number) per banana cultivation zones of Malawi. (DOCX) [file pone.0306671.s023.docx]

**S19 Table. Percentage (numbers) of detected BSV species per cultivation zone.** This S19 Table percentage (numbers) of detected BSV species (BSCAV, BSGFV, BSLAC, BSIMV, BSMYV and BSOLV) in percent (number) per banana cultivation zones of Malawi.

| Banana cultivation zones | Number of samples | Percentage (number) of BSV Positive to at least one of the following species: BSCAV, BSGFV, BSLAC, BSIMV, BSMYV and BSOLV. | | | | | | |
| --- | --- | --- | --- | --- | --- | --- | --- | --- |
|  |  | All BSV Species | BSCAV | BSGFV | BSIMV | BSLACV | BSOLV | BSMYV |
| Zone 1 | 70 | 27% (19) | 3% (2) | 8% (6) | 12% (9) | 0% (0) | 12% (9) | 1% (1) |
| Zone 2 | 66 | 20% (13) | 2% (1) | 0% (0) | 0% (0) | 2% (1) | 8% (5) | 6% (4) |
| Zone 3 | 65 | 14% (9) | 0% (0) | 5% (3) | 2% (1) | 0% (0) | 2% (1) | 6% (4) |
| Zone 4 | 74 | 28% (21) | 1% (1) | 15% (10) | 12% (8) | 0% (0) | 4% (3) | 16% (11) |
