# Supplementary material for: Surveillance and molecular characterization of banana viruses associated with Musa germplasm in Malawi
Source: PLoS One. 2026 Jan 29;21(1):e0306671. doi: 10.1371/journal.pone.0306671 (PMC12854425; doi:10.1371/journal.pone.0306671)
Supplement: S20 Table — Columns represent age of mat, number of mat, virus, prevalence (number), 95% CI = 95% confidence interval. *: significantly different (P < 0.05); ** very significantly different (P < 0.01); *** very highly significantly different (P < 0.001) and OR = odd number. (DOCX) [file pone.0306671.s024.docx]

**S20 Table. The effect of age of banana mats on prevalences of banana viruses: BBTV, BanMMV and BSV**. Columns represent age of mat, number of mat, virus, prevalence (number), 95% CI = 95% confidence interval. *: significantly different (P<0.05); ** very significantly different (P<0.01); very highly significantly different (P<0.001) and OR = odd number.

| Age of mat | Number of mats | Virus | Prevalence (n) | OR | 95% CI | P value |
| --- | --- | --- | --- | --- | --- | --- |
| 1-3 yrs | 107 | BBTV | 13 % (14) | Reference |  |  |
|  |  | BanMMV | 19 % (20) | Reference |  |  |
|  |  | BSV | 27 % (29) | Reference |  |  |
| 4-6 yrs | 38 | BBTV | 5 % (2) | 1.49 | 0.40 – 5.59 | 0.967 |
|  |  | BanMMV | 8 % (3) | 0.37 | 0.10 – 1.32 | 0.127 |
|  |  | BSV | 9 % (24) | 1.77 | 0.48–6.54 | 1.000 |
| Over 6 yrs | 130 | BBTV | 10 % (13) | 1.11 | 0.48 – 2.55 | 0.899 |
|  |  | BanMMV | 14 % (18) | 0.74 | 0.10 – 1.32 | 0.385 |
|  |  | BSV | 21 % (27) | 1.41 | 0.62–3.20 | 0.609 |
