# Supplementary material for: Surveillance and molecular characterization of banana viruses associated with Musa germplasm in Malawi
Source: PLoS One. 2026 Jan 29;21(1):e0306671. doi: 10.1371/journal.pone.0306671 (PMC12854425; doi:10.1371/journal.pone.0306671)
Supplement: S21 Table — Columns represent age of mat, number of mat, virus, prevalence (number), 95% CI = 95% confidence interval. *: significantly different (P < 0.05); ** very significantly different (P < 0.01); *** very highly significantly different (P < 0.001) and and OR = odd number. (DOCX) [file pone.0306671.s025.docx]

**S21 Table. The effect of banana cultivation systems on prevalences of banana viruses: BBTV, BanMMV and BSV.** Columns represent age of mat, number of mat, virus, prevalence (number), 95% CI = 95% confidence interval. *: significantly different (P<0.05); ** very significantly different (P<0.01); *** very highly significantly different (P<0.001) and and OR = odd number.

| Cultivation system | Number of mats | BBTV | BanMMV | BSV |
| --- | --- | --- | --- | --- |
| Mono cropping | 107 | 8 % (9) | 17 % (18) | 21 % (23) |
| Mixed cropping | 168 | 12 % (20) | 14 % (23) | 25 % (42) |
| P value |  | 0.382 | 0.678 | 0.625 |
| Odd number |  | 1.45 | 0.87 | 1.16 |
| 95% CI |  | 0.63 - 3.31 | 0.44 -  1.70 | 0.65 - 2.06 |
