# Supplementary material for: Surveillance and molecular characterization of banana viruses associated with Musa germplasm in Malawi
Source: PLoS One. 2026 Jan 29;21(1):e0306671. doi: 10.1371/journal.pone.0306671 (PMC12854425; doi:10.1371/journal.pone.0306671)
Supplement: S22 Table — Sequences generated from detected banana viruses in this study, segment analyzed and the assigned NCBI GenBank accession number. (DOCX) [file pone.0306671.s026.docx]

**S22 Table: Banana virus isolates from this study and their NCBI GenBank accession numbers.** Sequences generated from detected banana viruses in this study, segment analyzed and the assigned NCBI GenBank accession number.

| **Banana bunchy top virus** | | | **Banana mild mosaic virus** | | | **Banana streak virus** | | |
| --- | --- | --- | --- | --- | --- | --- | --- | --- |
| **Isolate** | **Segment** | **Accession number** | **Isolate** | **Segment** | **Accession number** | **Isolate** | **Segment** | **Accession number** |
| MWCK01 (MWCK02) | DNA-R | PP 796395 | MWJM-MJ21 | Coat | PP806141 | JMCK08 | Ribonuclease H | PP806157 |
| MWKK02 (MWKK02) | DNA-R | PP 796396 | MWJM-NB04 | Coat | PP806142 | JMKK02 | Ribonuclease H | PP806158 |
| MWKK03 (MWKK04) | DNA-R | PP 796397 | MWJM-KK01 | Coat | PP806143 | JMCK07 | Ribonuclease H | PP806159 |
| MWMJ04 (MWMJ17) | DNA-R | PP 796398 | MWJM-CK07 | Coat | PP806144 | JMCK18 | Ribonuclease H | PP806160 |
| MWNB05 (MWNB05) | DNA-R | PP 796399 | MWJM-CK05 | Coat | PP806145 | JMCP11a | Ribonuclease H | PP806161 |
| MWNB06 (MWNB20) | DNA-R | PP 796400 | MWJM-LL10 | Coat | PP806146 | JMCK05 | Ribonuclease H | PP806162 |
| MWPE07 (MWPE03) | DNA-R | PP 796401 | MWJM-LL13 | Coat | PP806147 | JMCK06 | Ribonuclease H | PP806163 |
| MWKK08 (MWKK03) | DNA-R | PP 796402 | MWJM-MJ11 | Coat | PP806148 | JMCP12 | Ribonuclease H | PP806164 |
| MWMJ09 (MWMJ12) | DNA-R | PP 796403 | MWJM-TO18 | Coat | PP806149 | JMCP11b | Ribonuclease H | PP806165 |
| MWNB10 (MWNB07) | DNA-R | PP 796404 | MWJM-DZ18 | Coat | PP806150 | JMDZ04 | Ribonuclease H | PP806166 |
| MWZA11 (MWZA16) | DNA-R | PP 796405 | MWJM-KK07 | Coat | PP806151 | JMDZ08 | Ribonuclease H | PP806167 |
| MWZA11 (MWZA18) | DNA-R | PP 796406 | MWJM-NB25 | Coat | PP806152 | JMKA02a | Ribonuclease H | PP806168 |
| MWKA12 (MWKA02) | DNA-R | PP 796407 | MWJM-NE06 | Coat | PP806153 | JMKA02b | Ribonuclease H | PP806169 |
|  |  |  | MWJM-KK10 | Coat | PP806154 |  |  |  |
|  |  |  | MWJM-NB09 | Coat | PP806155 |  |  |  |
|  |  |  | MWJM-PE13 | Coat | PP806156 |  |  |  |
