# Supplementary material for: Surveillance and molecular characterization of banana viruses associated with Musa germplasm in Malawi
Source: PLoS One. 2026 Jan 29;21(1):e0306671. doi: 10.1371/journal.pone.0306671 (PMC12854425; doi:10.1371/journal.pone.0306671)
Supplement: Attachment — Submitted filename: Responses to the Reviewers.docx [file pone.0306671.s029.docx]

Below is an improved and professionally edited version of your **point-by-point responses** to reviewers.
I preserved all scientific content and added clarity, precision, and appropriate academic tone.
You can copy/paste directly into your rebuttal document.
(If you would like, I can also format this into a clean tracked-changes Word file.)

**Revised Point-by-Point Responses to Reviewers**

**Reviewer #1**

**C1. Comment:**

*The methodology is valid overall; however, the lack of detail regarding sample selection and sample size, along with the absence of a power calculation, raises concerns about the reliability of the results.*

**A1. Response:**

We thank the reviewer for this insightful comment. Additional methodological detail has now been incorporated in several sections of the revised manuscript. Specifically, we have clarified the number of farms sampled per zone (45 farms per zone; 180 farms total) and the types of samples collected (lines 201–205, and 208–215).
We also added the number of individual leaf samples per zone and clarified that variations in sample numbers resulted from differences in cultivar diversity per farm (lines 344–348).
These additions improve the transparency and reliability of the sampling strategy.

**1. Comment: Limited sample size in some areas; need to justify sampling strategy.**

**A1 (second part). Response:**

As noted above, each of the four banana cultivation zones included 45 farms (180 total) (lines 201–202). We sampled all banana cultivars present at each farm, resulting in 275 individual leaf samples. Differences in sample numbers across zones reflect the natural variation in cultivar diversity at the farm level (lines 342–346).
This information has been explicitly added to better justify the sampling strategy.

**2. Comment: Potential sampling bias; more detail needed on how cultivars were sampled.**

**A2. Response:**

To reduce sampling bias, we employed the W-pattern sampling approach to generate a composite sample for each cultivar within farms containing multiple mats. Where only a single mat of a cultivar was present, that plant was sampled directly. These methodological details have been added to lines 203–207.

**3. Comment: Discussion of mixed infections should be expanded.**

**A3. Response:**

As recommended, we replaced “mixed infection” with “co-infection” throughout the manuscript. We expanded the discussion of co-infections and added a supplementary figure (S2 Fig) and table (S18 Table) detailing the specific sites where co-infections were observed. Additional explanations appear on lines 862–866.

**4. Comment: Absence of CMV and BBrMV should be more thoroughly explained.**

**A4. Response:**

We added clarification that CMV and BBrMV were not detected, likely due to their minimal or absent prevalence in Malawi (lines 857–858). This section has been expanded to provide additional context.

**5. Comment: Data availability should be more explicit.**

**A5. Response:**

A comprehensive dataset has been provided as Supplementary Table S5 as recommended. This table includes GPS location, district, banana cultivation zone, mat age, source of planting material, cultivar name, genotype, field survey sample ID, and IC-(RT)-PCR results for BanMMV, BSV, and BBTV, along with literature-based information on uncharacterized genotypes.

**Additional Improvement Points Recommended by Reviewer #1**

**1. Expand sampling methodology.**

**A. Response:**

Sampling methodology has been greatly expanded (lines 203–207, 208–218), including details on:

- the W-pattern sampling approach,
- sampling symptomatic vs asymptomatic plants,
- avoidance of fungal-infected leaves,
- sample handling procedures.

**2. Improve data presentation.**

**A. Response:**

We reorganized large tables into clearer, smaller ones and added new tables presenting statistical relationships between variables (S11–S17). Figures have been revised, and more detailed legends have been added. The discussion of co-infections has also been strengthened.

**3. Address study limitations.**

**A. Response:**

We expanded the limitations section to discuss detection sensitivity. Although alternative techniques exist, immunocapture RT-PCR remains the most reliable for banana tissues due to high tannin content. HTS (VANA) analysis on the same samples did not reveal additional viruses (Viruses 2025, 17(8), 1068). This strengthens the interpretation of virus absence in our dataset.

**4. Expand data availability.**

**A. Response:**

Raw underlying data are fully available in Supplementary Table S5 as requested.

**5. Strengthen discussion section.**

**A. Response:**

We expanded the discussion to highlight the implications for disease management, seed system formalization, and phytosanitary control (lines 886–893). These revisions clarify the practical relevance of our findings.

**Reviewer #2**

**General Comment:**

Reviewer #2 provided extensive in-text suggestions, which we have addressed.

**Response:**

All corrections suggested by Reviewer #2 have been implemented and highlighted in yellow in the revised manuscript. As requested:

- “sharing” was replaced with “exchange,”
- “mixed infection” with “co-infection,”
- zone identifiers (“z”) were standardized to uppercase “Z”.
  We appreciate Reviewer #2’s clear and constructive feedback.

**Reviewer #3**

**Q1. Comment: Data availability unclear; raw data not obviously accessible.**

**A1. Response:**

This comment has been fully addressed. All raw data underlying the study’s findings are now provided in Supplementary Table S5 and are explicitly cited in the Data Availability Statement.

**Q2. Comment: Statistical assumptions not described; need detail on tests performed.**

**A2. Response:**

We added results from Kolmogorov–Smirnov and Shapiro–Wilk tests (S2A–S2D Tables), as well as Snedecor and Cochran tests (S3A–S3E Tables).

**A3. Additional Response:**

A detailed subsection on normality testing has been added to the Methods (lines 283–286).
Effect sizes, including Phi coefficients (lines 289–290) and odds ratios for binomial analysis (lines 297–317), have also been incorporated.

**Ethical considerations were not explicitly stated.**

**A4. Response:**

We clarified that no ethical approval was required because:

- no personal or identifiable information was collected,
- only adults participated,
- interviews concerned non-personal information (cultivars, seed sources, disease knowledge).
  Verbal informed consent procedures have been added (lines 243–245).

**Discussion section lacked specificity regarding management strategies.**

**A5. Response:**

We expanded the discussion to include actionable recommendations for surveillance, formal seed system development, and enhanced phytosanitary measures (lines 888–889, 896–903).

**Minor Comments**

**Controls for PCR and sequencing insufficiently described.**

**A6. Response:**

We added information on the use of known infected reference materials from the University of Liege quarantine collection as positive controls, and PCR-grade water as a negative control (lines 250-252).

**Language and presentation issues.**

**A7. Response:**

The manuscript has been thoroughly proofread to improve clarity and accuracy.

**Figures and tables densely packed.**

**A8. Response:**

We have simplified and redesigned figures and tables, ensuring clearer legends and more accessible visual presentation. For Figures 1 and S2 captions, *“Map created by the authors”* was added and the‑source or public domain datasets; local online geodatabase MASDAP (Malawi Spatial Data Platform; <http://www.masdap.mw/catalogue/#/?f=dataset>) used has been added on lines 279-280.

**Sampling strategy representativeness insufficiently explained.**

**A9. Response:**

We clarified site selection criteria and the representativeness of the sampling framework (lines 203–211). Details on the W-pattern strategy and single-mat sampling have been strengthened.
