# Supplementary material for: Surveillance and molecular characterization of banana viruses associated with Musa germplasm in Malawi
Source: PLoS One. 2026 Jan 29;21(1):e0306671. doi: 10.1371/journal.pone.0306671 (PMC12854425; doi:10.1371/journal.pone.0306671)
Supplement: Peer review history

# Peer review: Decision Letter 0

**DOI:** 10.1371/journal.pone.0306671.r001

**Authors:** Osmond Ekwebelem

29 Jan 2025

Dear Dr. Masangwa,

Thank you for submitting your manuscript to PLOS ONE. After careful consideration, we feel that it has merit but does not fully meet PLOS ONE’s publication criteria as it currently stands. Therefore, we invite you to submit a revised version of the manuscript that addresses the points raised during the review process. Please find the reviewers' comments below this email.

Please submit your revised manuscript by Mar 15 2025 11:59PM. If you will need more time than this to complete your revisions, please reply to this message or contact the journal office at plosone@plos.org . When you're ready to submit your revision, log on to https://www.editorialmanager.com/pone/ and select the 'Submissions Needing Revision' folder to locate your manuscript file.

- A rebuttal letter that responds to each point raised by the academic editor and reviewer(s). You should upload this letter as a separate file labeled 'Response to Reviewers'.
- A marked-up copy of your manuscript that highlights changes made to the original version. You should upload this as a separate file labeled 'Revised Manuscript with Track Changes'.
- An unmarked version of your revised paper without tracked changes. You should upload this as a separate file labeled 'Manuscript'.

If you would like to make changes to your financial disclosure, please include your updated statement in your cover letter. Guidelines for resubmitting your figure files are available below the reviewer comments at the end of this letter.

If applicable, we recommend that you deposit your laboratory protocols in protocols.io to enhance the reproducibility of your results. Protocols.io assigns your protocol its own identifier (DOI) so that it can be cited independently in the future. For instructions see: https://journals.plos.org/plosone/s/submission-guidelines#loc-laboratory-protocols . Additionally, PLOS ONE offers an option for publishing peer-reviewed Lab Protocol articles, which describe protocols hosted on protocols.io. Read more information on sharing protocols at https://plos.org/protocols?utm_medium=editorial-email&utm_source=authorletters&utm_campaign=protocols .

We look forward to receiving your revised manuscript.

Kind regards,

Osmond Ekwebelem

Academic Editor

PLOS ONE

Journal requirements:

When submitting your revision, we need you to address these additional requirements.

1. Please ensure that your manuscript meets PLOS ONE's style requirements, including those for file naming. The PLOS ONE style templates can be found at

https://journals.plos.org/plosone/s/file?id=wjVg/PLOSOne_formatting_sample_main_body.pdf and

https://journals.plos.org/plosone/s/file?id=ba62/PLOSOne_formatting_sample_title_authors_affiliations.pdf.

2. In your Methods section, please provide additional information regarding the permits you obtained for the work. Please ensure you have included the full name of the authority that approved the field site access and, if no permits were required, a brief statement explaining why.

3. We note that the grant information you provided in the ‘Funding Information’ and ‘Financial Disclosure’ sections do not match.

When you resubmit, please ensure that you provide the correct grant numbers for the awards you received for your study in the ‘Funding Information’ section.

4. Thank you for stating the following financial disclosure:

[This work was supported by the European Union (EU) funded Development Smart Innovation through Research in Agriculture (DeSIRA) project.].

Please state what role the funders took in the study.  If the funders had no role, please state: ""The funders had no role in study design, data collection and analysis, decision to publish, or preparation of the manuscript.""

If this statement is not correct you must amend it as needed.

Please include this amended Role of Funder statement in your cover letter; we will change the online submission form on your behalf.

5. Please include captions for your Supporting Information files at the end of your manuscript, and update any in-text citations to match accordingly. Please see our Supporting Information guidelines for more information: http://journals.plos.org/plosone/s/supporting-information.

Reviewers' comments:

Reviewer's Responses to Questions

**Comments to the Author**

1. Is the manuscript technically sound, and do the data support the conclusions?

Reviewer #1: Yes

Reviewer #2: Yes

Reviewer #3: Yes

**********

2. Has the statistical analysis been performed appropriately and rigorously?

Reviewer #1: Yes

Reviewer #2: Yes

Reviewer #3: Yes

**********

3. Have the authors made all data underlying the findings in their manuscript fully available?

The PLOS Data policy

Reviewer #1: Yes

Reviewer #2: Yes

Reviewer #3: Yes

**********

4. Is the manuscript presented in an intelligible fashion and written in standard English?

Reviewer #1: Yes

Reviewer #2: Yes

Reviewer #3: No

**********

Reviewer #1: The results are highly valuable to Malawi and potentially other countries facing similar banana virus challenges. The findings regarding virus prevalence, genetic diversity, and the role of propagule sharing will inform effective disease management strategies. The characterization of unique Malawian germplasm is also valuable for breeding programs.

The writing is generally clear and concise, although some sentences could be improved for clarity and flow. The language is scientifically appropriate. Minor grammatical and stylistic edits would improve readability.

The methodology is valid overall. However, the lack of detail regarding sample selection and size, along with the absence of a power calculation to support the selected sample size, raises some concerns about the reliability of the results.

The results are generally discussed correctly but could be more comprehensive in terms of the level of detail provided. Addressing the weaknesses mentioned below would greatly improve the discussion.

This is a valuable contribution that addresses a crucial issue in banana production. Addressing the weaknesses identified would significantly enhance the manuscript's quality and impact. After these improvements are made, the research would be suitable for publication.

The valuable points of the study:

1. Addresses a significant problem: The research focuses on banana bunchy top disease (BBTD), a major threat to banana production in Malawi, providing valuable data for disease management.

2. Comprehensive methodology: The study employs a combination of field surveys, molecular characterization, and phylogenetic analysis, providing a multi-faceted approach.

3. Original data: The study provides original data on the prevalence and genetic diversity of banana viruses in Malawi, filling a knowledge gap.

4. Clear objectives and conclusions: The research questions are clearly stated, and the conclusions are well-supported by the findings.

5. Well-structured: The manuscript follows a logical structure and is generally well-organized.

However, the following points need to be considered for improvement or justification:

1. Limited sample size in some areas: The sample size from the four zones is not explicitly stated, and there is potential variability in the representation of the different zones. The description of the sampling strategy needs improvement to clarify the sampling approach used. If not possible, justify the choice you followed.

2. Potential bias in sampling: More detailed information is needed on the methodology for sampling banana cultivars to assess whether the selection represents the whole genetic diversity across the regions. More details of sample selection are required.

3. Discussion of mixed infections: The discussion about mixed infections could be more detailed. For example, clarifying the types of co-infections identified, their prevalence, and their potential impact on disease severity.

4. Absence of CMV and BBrMV: The absence of CMV and BBrMV could be elaborated more extensively to further explain its possible reasons and to offer some insight on the potential implications.

5. Data availability: While the authors state data availability, specific details on how the data will be made publicly accessible are lacking.

Improvement Points:

1. Expand on sampling methodology: Provide a more detailed explanation of the sampling strategy to ensure representativeness and reduce bias. Include information on sample size for each zone.

2. Improve data presentation: Include detailed tables and figures, and the supplementary tables should be mentioned explicitly in the manuscript and referenced in the relevant sections. For example, figures 1-4 need more information in the legends about what the reader can get from each figure. The figure should explain itself without the need to refer to the main text in the manuscript. Tables, should get more details as well. Also, enhance the discussion and analysis of mixed infections.

3. Address limitations: Thoroughly discuss the limitations of the study and how these might impact the interpretations. For example, is it possible to find the absent viruses if you use different methodology? Sampling? Varieties? Are there other viruses should be included in the future surveys? Even if they are not mainly affecting banana, but may enhance the infection of other main viruses.

4. Expand on data availability: Clearly explain how the data underlying the findings will be made available to the wider scientific community.

5. Enhance the discussion section: This section should be expanded to more fully elaborate on the findings' implications for disease management and breeding.

Reviewer #2: The study was conducted to evaluate the presence and incidence of banana viruses in Malawi. A nationwide disease survey was conducted to identify banana viruses through targeted molecular characterization and analyze their association with banana genotypes, seed sources, cropping system, and age of plantation. The authors well implemented the research, analyzed the data, and reported. The results have regional importance in the efforts to eradicate notorious viral diseases in Southern Africa. A detailed review comment was provided in the attached manuscript.

Reviewer #3: Dear Authors,

Your manuscript "Surveillance and molecular characterization of banana viruses and their association with Musa germplasm in Malawi" presents a significant study that addresses crucial aspects of agricultural virology in Malawi. The subject is highly relevant, and your findings could have substantial implications for banana cultivation management and disease mitigation strategies. However, there are several areas where improvements are necessary to meet the publication standards of PLOS ONE.

MAJOR COMMENTS

-Data Availability and Transparency (General throughout the manuscript)

Major Issue: You mention that all relevant data are within the manuscript and its Supporting Information files. However, it is unclear whether the raw data points behind the statistical summary (means, medians, variance) are fully accessible.

Action Required: Please clarify this in the Data Availability section and consider depositing all raw data in a public repository, providing direct access links within the manuscript.

-Statistical Analysis (Results section)

Major Issue: The manuscript employs chi-square tests and ANOVA; however, the assumptions of these tests (such as normality and homogeneity of variance for ANOVA) are not discussed.

Action Required: Please add a subsection in the Methods detailing these assumptions and any tests performed to verify them. Also, include effect sizes in your results to provide insights into the magnitude of observed effects, which is crucial for practical application of your findings.

-Ethical Considerations (Methods section)

Major Issue: The study involves direct interaction with farmers through surveys, but there is no explicit mention of ethical approval from an institutional review board or similar body.

Action Required: Clarify the ethical review process and include details on informed consent, ensuring compliance with international standards for research ethics.

-Implications for Future Research and Practical Application (Discussion section)

Major Issue: The discussion on management strategies based on your findings is vague and lacks specificity.

Action Required: Expand this section to propose specific, actionable strategies for managing banana viruses in Malawi, possibly suggesting future lines of research that could stem from your findings.

MINOR COMMENTS

-Methodological Details (Methods section)

Minor Issue: The control measures used in PCR and sequencing experiments are not adequately described, which could affect reproducibility.

Action Required: Please provide detailed descriptions of the controls, including any reference materials or standards used.

-Language and Presentation (Throughout the manuscript)

Minor Issue: There are several instances of awkward phrasing and typographical errors that could hinder comprehension and distract readers.

Action Required: Conduct a thorough proofreading session to correct these issues. Consider engaging a professional language editing service if necessary.

Minor Issue: Figures and tables are densely packed with data, making them difficult to interpret.

Action Required: Simplify these visual elements and provide clearer, more descriptive legends and labels.

-Sampling Strategy (Methods section)

Minor Issue: The representativeness of the sampling strategy across different zones in Malawi is asserted but not substantiated with supporting data or rationale.

Action Required: Elaborate on how the sites were selected and any steps taken to ensure a representative cross-section of Malawi’s banana cultivation areas.

**********

what does this mean? ). If published, this will include your full peer review and any attached files.

If you choose “no”, your identity will remain anonymous but your review may still be made public.

**Do you want your identity to be public for this peer review?** For information about this choice, including consent withdrawal, please see our Privacy Policy

Reviewer #1: **Yes:**  Mustafa Adhab, Department of Plant Protection, University of Baghdad

Reviewer #2: **Yes:**  Amelework B Assefa

Reviewer #3: No

**********

[NOTE: If reviewer comments were submitted as an attachment file, they will be attached to this email and accessible via the submission site. Please log into your account, locate the manuscript record, and check for the action link "View Attachments". If this link does not appear, there are no attachment files.]

While revising your submission, please upload your figure files to the Preflight Analysis and Conversion Engine (PACE) digital diagnostic tool, https://pacev2.apexcovantage.com/ . PACE helps ensure that figures meet PLOS requirements. To use PACE, you must first register as a user. Registration is free. Then, login and navigate to the UPLOAD tab, where you will find detailed instructions on how to use the tool. If you encounter any issues or have any questions when using PACE, please email PLOS at figures@plos.org . Please note that Supporting Information files do not need this step.

Attachment Submitted filename: PONE-D-24-22970 with reviewers comment.pdf

# Author comment: Author response to Decision Letter 1

**DOI:** 10.1371/journal.pone.0306671.r002

14 Dec 2025

This article has been revised as such it has some additional supporting new tables and figure. Enrichment on materials and methods section has also been done.

Attachment Submitted filename: Responses to the Reviewers.docx

# Editor report: Decision Letter 1

**DOI:** 10.1371/journal.pone.0306671.r003

**Authors:** Osmond Ekwebelem

21 Dec 2025

Surveillance and molecular characterization of banana viruses associated with Musa germplasm in Malawi

PONE-D-24-22970R1

Dear Mr. Masangwa,

We’re pleased to inform you that your manuscript has been judged scientifically suitable for publication and will be formally accepted for publication once it meets all outstanding technical requirements.

Within one week, you’ll receive an e-mail detailing the required amendments. When these have been addressed, you’ll receive a formal acceptance letter and your manuscript will be scheduled for publication.

An invoice will be generated when your article is formally accepted. Please note, if your institution has a publishing partnership with PLOS and your article meets the relevant criteria, all or part of your publication costs will be covered. Please make sure your user information is up-to-date by logging into Editorial Manager at Editorial Manager®  and clicking the ‘Update My Information' link at the top of the page. For questions related to billing, please contact billing support .

If your institution or institutions have a press office, please notify them about your upcoming paper to help maximize its impact. If they’ll be preparing press materials, please inform our press team as soon as possible -- no later than 48 hours after receiving the formal acceptance. Your manuscript will remain under strict press embargo until 2 pm Eastern Time on the date of publication. For more information, please contact onepress@plos.org.

Kind regards,

Osmond Ekwebelem

Academic Editor

PLOS One

Additional Editor Comments (optional):

Reviewers' comments:

# Editor report: Acceptance letter

**DOI:** 10.1371/journal.pone.0306671.r004

**Authors:** Osmond Ekwebelem

PONE-D-24-22970R1

PLOS One

Dear Dr. Masangwa,

I'm pleased to inform you that your manuscript has been deemed suitable for publication in PLOS One. Congratulations! Your manuscript is now being handed over to our production team.

At this stage, our production department will prepare your paper for publication. This includes ensuring the following:

* All references, tables, and figures are properly cited

* All relevant supporting information is included in the manuscript submission,

* There are no issues that prevent the paper from being properly typeset

You will receive further instructions from the production team, including instructions on how to review your proof when it is ready. Please keep in mind that we are working through a large volume of accepted articles, so please give us a few days to review your paper and let you know the next and final steps.

Lastly, if your institution or institutions have a press office, please let them know about your upcoming paper now to help maximize its impact. If they'll be preparing press materials, please inform our press team within the next 48 hours. Your manuscript will remain under strict press embargo until 2 pm Eastern Time on the date of publication. For more information, please contact onepress@plos.org.

You will receive an invoice from PLOS for your publication fee after your manuscript has reached the completed accept phase. If you receive an email requesting payment before acceptance or for any other service, this may be a phishing scheme. Learn how to identify phishing emails and protect your accounts at https://explore.plos.org/phishing.

If we can help with anything else, please email us at customercare@plos.org.

Thank you for submitting your work to PLOS ONE and supporting open access.

Kind regards,

PLOS ONE Editorial Office Staff

on behalf of

Dr. Osmond Ekwebelem

Academic Editor

PLOS One
